# Hierarchical Uniform Supramolecular Conjugated Spherulites with Suppression of Defect Emission

**DOI:** 10.1016/j.isci.2019.06.002

**Published:** 2019-06-08

**Authors:** Changjin Ou, Nathan J. Cheetham, Jiena Weng, Mengna Yu, Jinyi Lin, Xuhua Wang, Chen Sun, Juan Cabanillas-Gonzalez, Linghai Xie, Lubing Bai, Yamin Han, Donal D.C. Bradley, Wei Huang

**Affiliations:** 1 School of Physical and Mathematical Sciences & Institute of Advanced Materials (IAM), Jiangsu National Synergetic Innovation Center for Advanced Materials (SICAM), Nanjing Tech University (NanjingTech), 30 South Puzhu Road, Nanjing 211816, China; 2 Center for Molecular Systems and Organic Devices (CMSOD), Key Laboratory for Organic Electronics and Information Displays & Institute of Advanced Materials (IAM), Jiangsu National Synergetic Innovation Center for Advanced Materials (SICAM), Nanjing University of Posts & Telecommunications, 9 Wenyuan Road, Nanjing 210023, China; 3 Department of Physics and Centre for Plastic Electronics, The Blackett Laboratory, Imperial College London, Prince Consort Road, London SW7 2AZ, UK; 4 Shaanxi Institute of Flexible Electronics (SIFE), Northwestern Polytechnical University (NPU), 127 West Youyi Road, Xi'an 710072, Shaanxi, China; 5 Madrid Institute for Advanced Studies (IMDEA Nanociencia), Ciudad Universitaria de Cantoblanco, Calle Faraday 9, Madrid 28049, Spain; 6 Departments of Engineering Science and Physics and Division of Mathematical, Physical and Life Sciences, University of Oxford, 9 Parks Road, Oxford OX1 3PD, UK

**Keywords:** Optical Materials, Optics, Optoelectronics, Supramolecular Chemistry

## Abstract

Easily processed, well-defined, and hierarchical uniform artificial architectures with intrinsic strong crystalline emission properties are necessary for a range of light-emitting optoelectronic devices. Herein, we designed and prepared ordered supramolecular spherulites, comprising planar conformational molecules as primary structures and multiple hydrogen bonds as physical cross-links. Compared with serious aggregation-induced fluorescence quenching (up to 70%), these highly ordered architectures exhibited unique and robust crystalline emission with a high PLQY of 55%, which was much higher than those of other terfluorenes. The primary reasons for the high PLQY are the uniform exciton energetic landscape created in the planar conformation and the highly ordered molecular packing in spherulite. Meanwhile, minimal residual defect (green-band) emissions are effectively suppressed in our oriented crystalline framework, whereas the strong and stable blue light radiations are promoted. These findings may confirm that supramolecular ordered artificial architectures may offer higher control and tunability for optoelectronic applications.

## Highlights

• Coplanar molecular conformation is stabilized in supramolecular crystalline frameworks
• Spiro-terfluorene can self-assemble into hierarchical well-defined spherulites
• Ordered and uniform condensed structures suppress defect emission

## Graphical Abstract

**Figure:**
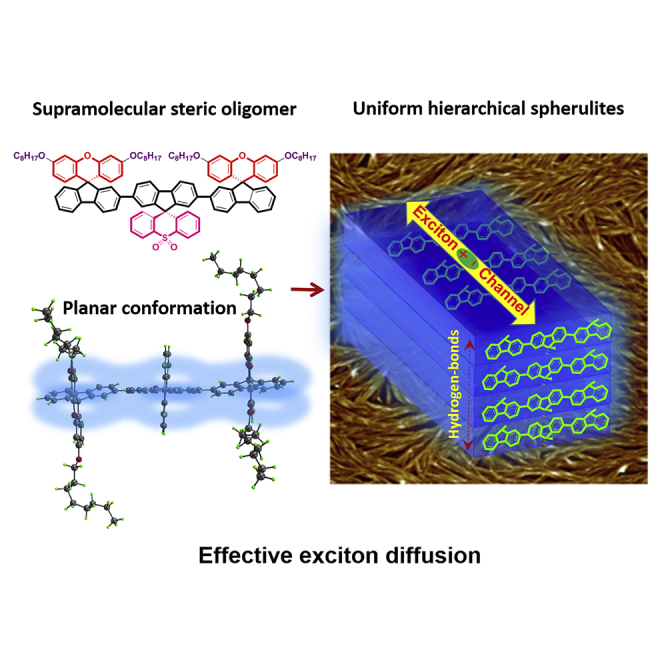

## Introduction

Construction of ordered organic artificial architectures is fundamental to the exploration of novel material systems in the disruptive technology area, and some have shown promising applications as functional and active units in electronic, smart, and biosystems, owing to their unprecedented properties and intriguing functions (Hoeben et al., 2005, Liu et al., 2015, Sanchez et al., 2005, van de Burgt et al., 2017, Zhang et al., 2016). Natural systems provide numerous examples of precise and efficient self-assembly processes, such as DNA functions of replication and transcription in biological systems and polypeptide structures as highly ordered, multilevel, condensed, complex macromolecules capable of highly specific functionalities (Costa et al., 2013, Wang et al., 2016). Fundamentally, the dynamic and reversible nature of orthogonal hydrogen-bonding interactions is an intrinsic, original, and basic factor enabling these organized macromolecules to self-assemble into advanced supramolecular functional systems (Aida et al., 2012, Hoeben et al., 2005, Lehn, 2010, Sengupta and Würthner, 2013). Compared with the conventional superstructure with a range of common smart and mechanical properties, supramolecular conjugated solid states display a wide variety of complicated, photophysical mechanisms for electron delocalization, hybridization, and coupling (Heeger, 2010, Huang et al., 2019, Liu et al., 2017a, Liu et al., 2017b; Mikhnenko et al., 2015, Xie et al., 2012, Yang et al., 2018, Ye et al., 2018). Similar to the ordered, natural systems, the highly uniform and well-defined conjugated nanostructures that are receptive to processing can provide an effective and favorable electronic micro-environment that promotes exciton diffusion (Jin et al., 2018, Lin et al., 2010, Mikhnenko et al., 2015) and energy transport (Haedler et al., 2015, Kim et al., 2013, Vogelsang et al., 2011). An ideal environment for the photophysical processes without irregularities plays a key role to a range of functions for organic devices (Venkateshvaran et al., 2014, Yan et al., 2009).

**Scheme 1:**
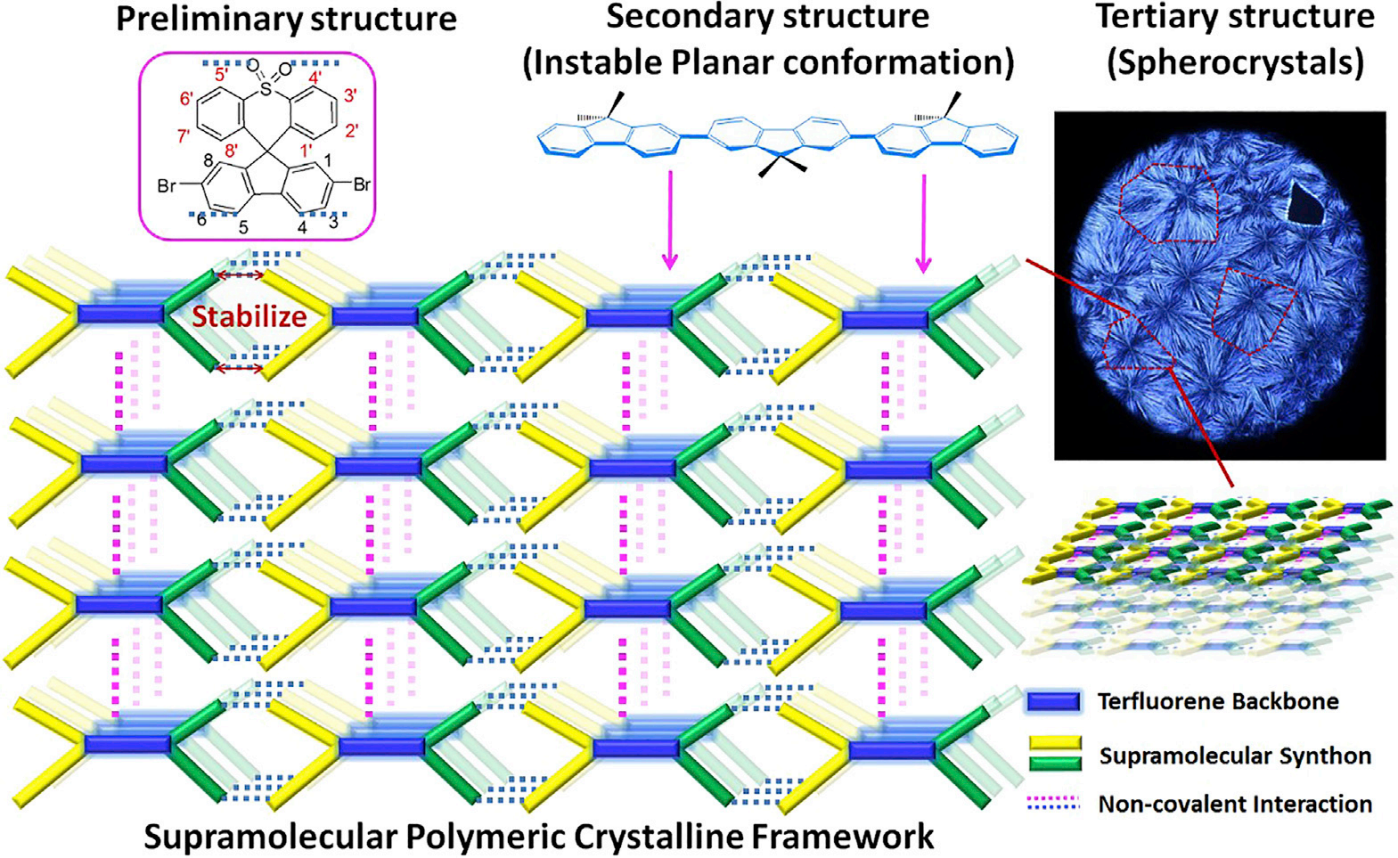
Schematic Illustration of Hierarchical Highly Ordered Condensed Structures of Synthetic Organic Semiconductors: Steric Molecular Structures, Oriented Planar Conformation, and Uniform Supramolecular Spherulites

In printed optoelectronics, the orderly condensed structure of a solution-processed π-conjugated semiconductor consistently optimized the fundamental photophysical processing of the active thin film, thereby enhancing the device performance (Eisele et al., 2014, Lunt et al., 2010, Mikhnenko et al., 2015, Peumans et al., 2003). For instance, ultralong exciton diffusion lengths (up to >200 nm) (Jin et al., 2018), energy transport lengths (>2 μm), and outstanding charge mobility (100 cm^2^ V^−1^ s^−1^) (Li et al., 2017, Yan et al., 2009) are obtained in well-defined supramolecular artificial architectures. To optimize this photophysical property, the chemical and physical defects should be avoided in solid states. Beyond chemical impurity and structural defects, intrinsic physical defects with an energetic disorder can constrain exciton combination or diffusion and block energy transfer or charge transport (Barford and Duffy, 2006, Noriega et al., 2013, Podzorov, 2013). Until now, uniform condensed structures are associated with tertiary structures, defined as regular and periodic molecular packing and arrangement of conjugated molecules. Compared with stable amorphous states, the ordered, uniform superstructures with simultaneously oriented conformations at molecular levels are energetically unstable states. These can only be stabilized in noncovalent interactions, assisted by hierarchical architectures (Haedler et al., 2015, Liu et al., 2017a, Liu et al., 2017b). The rigid π-conjugated backbone structure with its highly molecular orientation and order presents the following challenges: first, it possesses complicated and variable conformations and high-energy features and second, it is polymorphous in solid state and characterized by several competitive weak intra- and intermolecular interactions (Kim et al., 2013). In addition, molecular conformation can be precisely controlled by noncovalent interactions to stabilize the intrinsic steric energy generated by the formation of ordered and oriented conformations and phases in higher-level superstructures (Kim et al., 2013, Liu et al., 2017a, Liu et al., 2017b). In fact, dynamic noncovalent bonds can easily maintain and release energy, providing the important feature of allowing energy dissipation under strain, and subsequently recovering to achieve high stretchability and auto-recovery properties (Chen et al., 2012, Oh et al., 2016, Wu et al., 2008, Yang and Urban, 2013). With this in mind, to simulate the natural, multilevel superstructure induced by the noncovalent interaction (Hoeben et al., 2005, Sengupta and Würthner, 2013), the incorporation of dynamic crosslinking noncovalent bonds into supramolecular architectures is an effective and simple method to obtain both oriented and ordered conformation, and higher-level condensed states (Scheme 1).

**Figure 1:**
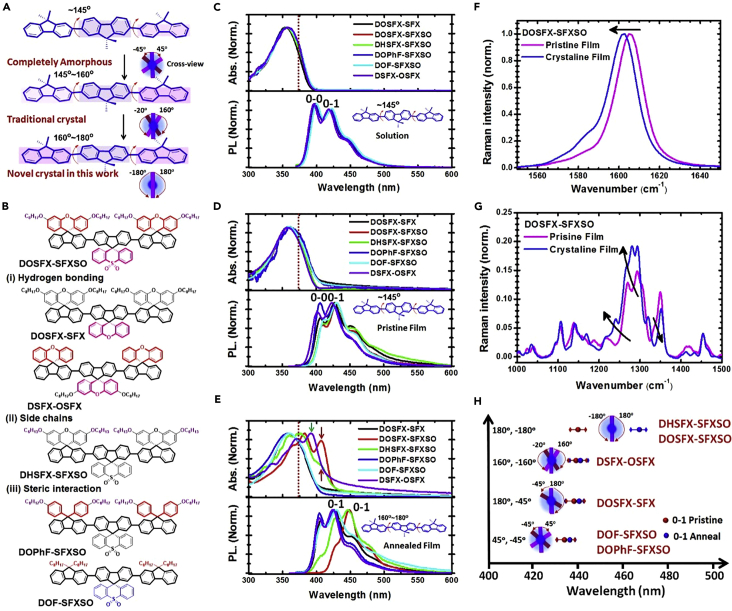
Molecular Structures, Photophysical Properties and Raman Spectra of Spiro-Terfluorenes (A) Conformational transition and polymorphic behavior of fluorene-based semiconductors: completely amorphous state (∼-45°), crystalline domain with nonplanar conformation (145°∼160°), and crystalline domain with an oriented planar conformation (160°∼180°). A schematic illustration is also shown here to screen fluorene units from cross view (red, purple, and pink plates represent fluorene monomers along backbone structures). (B) Chemical structures of terfluorene in this work. (C) Absorption and emission spectra of spiro-functionalized terfluorenes in dilute dichloromethane solution (10^−5^ mol/mL). (D) Absorption and emission spectra of pristine films of spiro-functionalized terfluorenes spin-coating from 10 mg/mL in toluene solution. (E) Absorption and emission spectra of annealed films of spiro-functionalized terfluorenes. Both DOSFX-SFXSO and DHSFX-SFXSO were annealed at 160°C. (F and G) Raman spectra of pristine spin-coated films and annealed films of DOSFX-SFXSO in the region of 1550∼1650 (F) and 900∼1500 (G) cm^−1^, respectively. (H) 0-1 Peaks of our fluorene-based six trimer molecule annealed films, together with their cross-sectional views of various conformations.

In this study, we established a molecular design principle of supramolecular conjugated macromolecules with orthogonal hydrogen-bonding interactions to enable a secondary planar conformation toward controlling and improving photonic behavior, defined as supramolecular plastic photonics (SPPs: photonic behavior of polymeric system can be significantly improved by supramolecular approach). This oriented planar conformation self-organizes into ordered tertiary microstructure supramolecular spherulites in accordance with the synergistically molecular attractor-repulsor theory (Scheme 1, Figures 1A and 1B) (Li et al., 2018, Wang et al., 2018, Xu et al., 2018). A hierarchical supramolecular artificial architecture with a series of multidimensional orthogonal hydrogen bonds in rigid crystalline framework is obtained from our bulky supramolecular spiro-terfluorene, 2,7-bis(3′,6′-bis(octyloxy)spiro[fluorene-9,9′-xanthen]-2-yl)spiro[fluorene-9,9′-thioxanthene]-10′,10′-dioxide (DOSFX-SFXSO). As a result of the confinement and stabilization of the rigid supramolecular framework, the molecules show an unusual unstable planar conformation in crystalline states. Interestingly, as expected, this planar conformation also enables molecules to self-assemble into higher-order condensed structures, namely, spherulites, rarely reported in organic electronics for optoelectronic devices. In contrast to conventional sulfur-containing conjugated molecules (Hoeben et al., 2005, Jin et al., 2018), our supramolecular terfluorene has an unusual suppression defect emission, which DOSFX-SFXSO can maintain at a high photoluminescent (PL) quantum efficiency of 55% under well-defined crystalline structures. Robust and stable excitonic behavior without any exciton trapped defect was observed for the supramolecular spherulites and explained this outstanding property.

## Results and Discussion

### Rational Design of Steric Terfluorenes

As shown in Figure 1, six different terfluorenes sharing the same conjugated backbone were designed and synthesized. First, we show the three important design features on the chemical structure of DOSFX-SFXSO in Figure 1B as follows: a centered spirofluorene unit SFXSO to induce the orthogonal hydrogen-bonding interaction, steric bulky units at 9-position of side fluorene to inhibit intermolecular stacking, and bulky side chains preventing side chain interdigitation. To date, as the most important component of bulky spiro-building blocks, the SFX unit is an effective steric group that suppresses intermolecular interactions. The difference between DOSFX-SFXSO and both 2′,7′-bis(9,9-bis(4-(octyloxy)phenyl)-9H-fluoren-2-yl)spiro[fluorene-9,9′-thioxanthene]-10′,10′-dioxide (DOPhF-SFXSO) and 2′,7′-bis(9,9-dioctyl-9H-fluoren-2-yl)spiro[fluorene-9,9′-thioxanthene]-10′,10′-dioxide (DOF-SFXSO) is the bulky steric unit on 9-position of the side fluorene units, which is necessary to investigate the steric interaction and the noncovalent interactions induced by the alkoxy chain. Frequently, noncovalent interactions among H, S, F, and O are useful to stabilize planar conformations and enhance crystallinity (Kim et al., 2013). According to our previous works, diverse and strong hydrogen-bonding interactions were observed in the SFXSO units (S=O···H-C), which could confer photophysical properties in aggregate state (Ou et al., 2017a, Ou et al., 2017b). Therefore, DOSFX-SFX was designed and prepared to determine the effect of this promising noncovalent interaction on conformation and crystalline properties. Furthermore, to explore the effect of the length of the substituted side chain, DHSFX-SFXSO with hexyl chains was prepared. The length of the alkoxy chain will precisely tune the molecular and chain packing in solid states (Liu et al., 2017a, Liu et al., 2017b). All materials were synthesized by Suzuki-type reaction with yield over 70%, and the detailed synthetic procedures and structure characterization are showed in Schemes S1 and S2 and Figures S28–S49. As showed in Figure S1 and Table S1, the on-set oxidation potential of terfluorene derivatives is around 0.86 to 0.98 V, so the highest occupied molecular orbital is around −5.62 to −5.72 eV. Similarly, the lowest unoccupied molecular orbital is around −2.54 to −2.69 eV and the bang gap is around 3.0 eV. The results indicate that nonconjugated electron-withdrawing sulfone groups have little influence on frontier orbitals. As expected, six terfluorenes have similar absorption and emission profiles, and only one absorption band with a maximum absorption peak of ∼355 and ∼360 nm, respectively, in both solution and pristine spin-coated films (Figures 1C and 1D), owing to a similar conjugated backbone structure. In addition, their emission spectra exhibit three well-resolved emission bands. The results indicate that the substitutions have a small influence on the electronic structures of terfluorene backbones. In contrast to the unchanged optical property of other controlled terfluorenes, DOSFX-SFXSO, DHSFX-SFXSO, and DSFX-OSFX show dramatic differences in the absorption and emission spectra of solid films after thermal treatment (Figure 1E). The well-structured absorption spectra of the annealed films of DOSFX-SFXSO and DHSFX-SFXSO have peaks at 382 and 379 nm, respectively, with the same shoulder absorption peak at approximately 406 nm (Figure 1E). Similarly, the maximum absorption peak of DSFX-OSFX is red-shifted from 357 to 390 nm. The above results indicate that molecular conformations are ordering and planarizing in the annealed film, resulting in a longer effective conjugation length (Bai et al., 2017, Ou et al., 2017a, Yu et al., 2018). Besides, the maximum emission peaks are bathochromic from 430 to 448 nm. In fact, the spectral changes are not complete in DHSFX-SFXSO. When the temperature was elevated from 160°C to 180°C, DOSFX-SFXSO and DHSFX-SFXSO displayed identical absorption and emission profiles (Figure S12). On the basis of our previous results about fluorene dimers and polymers, we can attribute these absorption and PL spectral shifts to conformational planarization of conjugated backbones.

Raman measurement is an effective tool to check the molecular conformational transition (Figures 1F and 1G) (Yu et al., 2018). Different aggregate states of DOSFX-SFXSO films were studied by Raman measurement to explain the molecular conformation and phase behavior. As indicated in Figures 1F–1H, the C=C stretching modes within spirofluorene units are bathochromic from 1,606 cm^−1^ of the amorphous phase to 1,602 cm^−1^ of the crystalline phase, which is attributable to the increased delocalized degree of π-electrons from a twisted conformation to a planar one. Compared with the amorphous state, the intensity ratio of the peaks 1,241, 1,266, 1,281, and 1,285 cm^−1^ to 1,602 cm^−1^ are increasing obviously in the crystalline phase. In addition, the vibrational frequency of the C-C stretching modes between spirofluorenes is hypochromatic from 1,349 cm^−1^ of the amorphous phase to 1,352 cm^−1^ of the crystalline phase. All the above Raman motions are similar to β-conformation in poly(9,9-dioctylfluoren-2,7-diyl) (PFO) and poly[4-(octyloxy)-9,9-diphenylfluoren-2,7-diyl]-co-[5-(octyloxy)-9,9-diphenylfluoren-2,7-diyl] (PODPF) (Liu et al., 2016, Liu et al., 2017a, Liu et al., 2017b, Yu et al., 2018). In this regard, we have further confirmed the formation of planar conformation in our DOSFX-SFXSO crystalline films.

**Figure 2:**
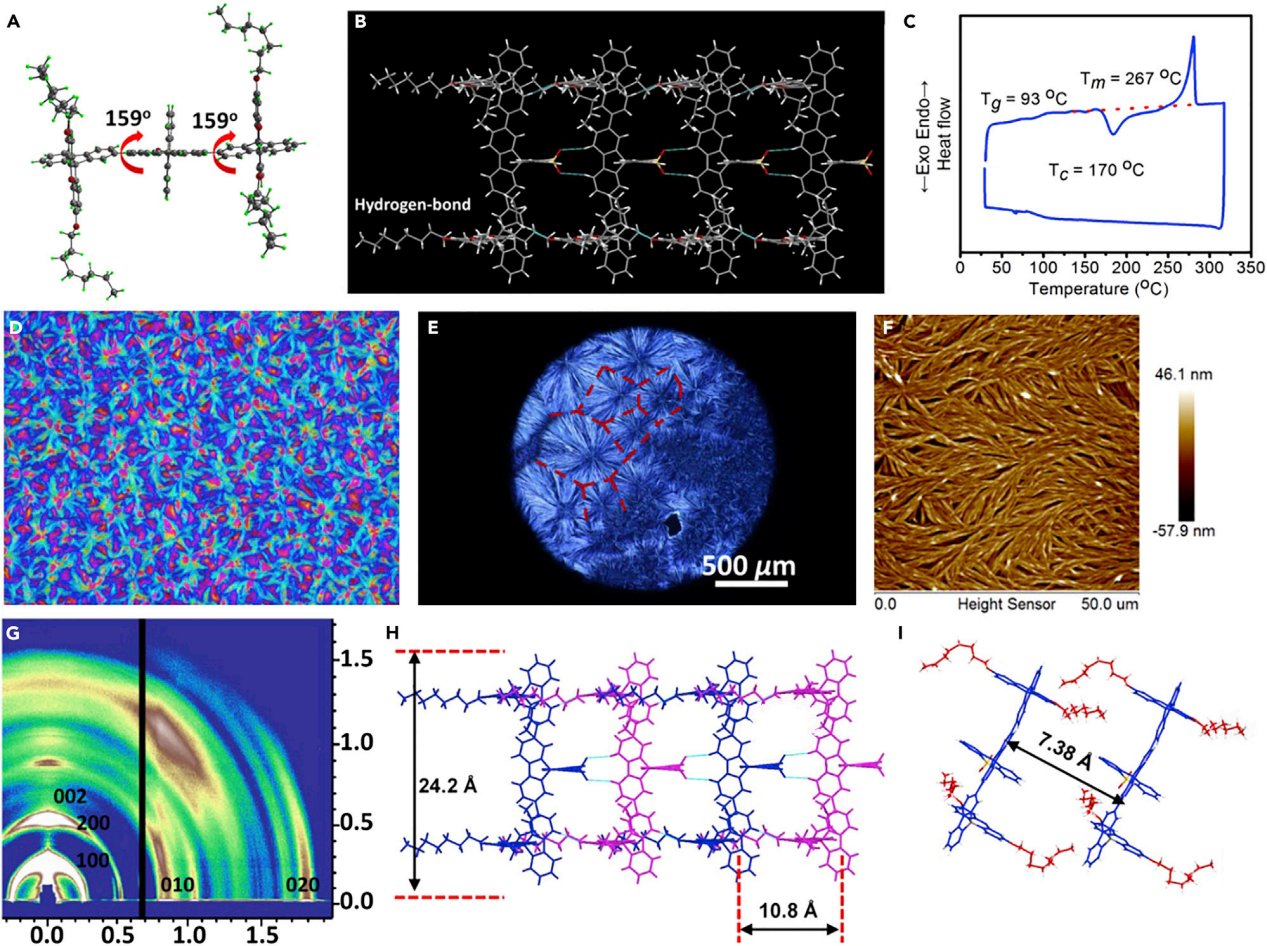
Crystal Structure and Packing Pattern, Thermal Behavior, Film Morphologies, and X-ray Diffraction of DOSFX-SFXSO (A) Crystallographic structure of DOSFX-SFXSO. (B) DOSFX-SFXSO-based hydrogen-bonded supramolecular polymer in crystalline framework. (C) Differential scanning calorimetric curve of DOSFX-SFXSO. (D and E) (D) Polarized optical and (E) cross-polarized optical micrographs of DOSFX-SFXSO annealed films. (F) Atomic force microscopic image of DOSFX-SFXSO nanofiber in spherulites. (G) Grazing-incident X-ray diffraction pattern image of DOSFX-SFXSO annealed films. (H and I) Schematic representation of the molecular packing in DOSFX-SFXSO nanofibers. (H) The length of conjugated backbone and intermolecular distance. (I) The distance intermolecular π-π interaction.

To verify the assumption above, we tried to cultivate the single crystals of our six materials (Figures 2A and S2–S11, and Table S2). Interestingly, two types of DOSFX-SFXSO single crystals were obtained: flake and needle (Figure S2). Needle crystal of DOSFX-SFXSO displays two absorption peaks around 382 and 407 nm and red-shifted emission (Figure S13), which is consistent with the results of annealing films, whereas the absorption spectra of flake crystals consist of peaks at 360 nm and 387 nm. As shown in Figure 2A, the DOSFX-SFXSO flake single crystal adopts a planar conformation with the same torsional angles of 159°. Owing to the large shift in absorption between the two crystals (∼20 nm), the molecular conformation in needle crystals is more planar and torsional angles can be close to 180°. Furthermore, DHSFX-SFXSO also has two crystal polymorphs: needle and rod, and they display different PL profiles (Figure S14). The rod crystal presents a bent and *trans*-*cis* conformation with torsional angles of −29.0° and −146.1° (Figure S3). In addition, the quasi-planar conformation of DOSFX-SFXSO and the twisted conformation of DHSFX-SFXSO could also transform into fully coplanar conformations after thermal treatment at 160°C (Figures S15 and S16). As expected, DOF-SFXSO adopts a *trans-trans* conformation with large torsional angles of −138.3° and −142.0° (Figure S3). In a word, various absorption and PL spectra of our six materials also present different conformations and 0-1 emission peaks in annealed films (Figure 1H).

As displayed in Figures 2A and 2B, supramolecular hydrogen-bonding interactions between DOSFX-SFXSO molecules with characteristic distances of 2.639 Å (S=O···H-C between sulfone group, and 4,5-position hydrogen atoms of the centered fluorene unit) and 2.385 Å (C-O···H-C between O atom of octoxyl chains and 4,5-position hydrogen atoms of adjacent fluorene unit) suggest the formation of a DOSFX-SFXSO-based supramolecular polymeric framework in single crystal. The hydrogen bonds can restrict intramolecular rotation and trap coplanar conformations of DOSFX-SFXSO. In other words, the steric hindrance of SFX and SFXSO render DOSFX-SFXSO to separate from each other. In this way, the molecule may have a large space to freely rotate. Then, the conformation is locked by hydrogen bonding of SFXSO, and the van der Waals forces of alkyl chains also take part in this process (Figure 2B). The hydrogen bonds involved in 4,5-position hydrogen atoms of SFXSO and the fluorene group promote DOF-SFXSO to form dimers with π-π stacking (3.786 Å), which impede intramolecular rotation. As a result, DOF-SFXSO adopts a highly twisted conformation. Therefore, the orthogonal hydrogen bonds in our supramolecular framework can not only stabilize the planar conformation but also obtain higher self-organized crystalline structures in a condensed structure.

Upon combining the crystal data with the results of photophysics, we make three key observations. First, the small chemical modification in DOSFX-SFXSO with DOSFX-SFX leads to significant differences in conformational behavior, clearly indicating that the supramolecular steric hindrance (SSH) effect of SFXSO group plays a key role in conformational planarization. Second, replacing OSFX groups with 9,9-dioctylfluorenyl and 9,9-dioctoxyphenylfluorenyl groups demonstrates that the spiro-configuration is essential to conformational planarization. Finally, both hexoxyl and octoxyl substitutions can induce conformational planarization in crystals and annealed films, whereas hexoxyl substitution maintains a higher thermal annealing temperature, an indication that the length of alkyl chain also influences molecular conformation and packing.

### Construction and Morphological Structure of Steric Terfluorenes-Based Supramolecular Spherulites

According to the differential scanning calorimetric curve in Figure 2C, a phase transformation (thermal-induced crystallization) of DOSFX-SFXSO occurs at 170°C and gives off a lot of latent heat (ΔQc ≈ −41.8 kJ/mol), which is slightly less than the heat of fusion (ΔHm ≈ 49.6 kJ/mol). Interestingly, if we kept a DOSFX-SFXSO pristine spin-coated film on a hot plate, we could see large supramolecular spherulites of several micrometers forming on the substrates (Figures 2D–2F and S17–S24). Besides, DSFX-OSFX crystalline structure could also be obtained after thermal annealing (Figure S25). Similar to conventional ones, our spherulites consisted of a centered crystal nucleus and a range of nanowires with an average width of ∼100 nm and height of ∼20 nm. As shown in Figures 2D and S17–S23, a large variation in spherulite semicrystalline was observed with domain size, from <10 up to 600 μm for edge-to-edge domain diameters. In fact, the domain size also decreases with increasing growth speed of crystal, indicating that slow crystallization promotes the molecular rearrangement and adjusts to the optimal packing position (Figure S23). Meanwhile, we also investigated the growth processing of spherulite at different thermal annealing temperatures ranging from 160°C to 186°C (Figure S22). First, no crystal was formed from 160°C to 168°C. Next, we obtained some small spherulites (∼10 μm) at 172°C, but the growth rate was very slow. Finally, the growth rate was significantly enhanced when the temperature rose above 182°C. In this regard, the growth rate was significantly dependent on the annealing temperature with a critical threshold of 168°C. Different colors observed in Figure 2D indicate various molecular orientations in well-defined spherulites (Figures S20 and S21). We can postulate that the dark areas on the film surface might be located in lower areas or occupied relatively disordered amorphous regions. We can see very clearly the boundaries between different spherulite semicrystalline domains. To further uncover the molecular packing and arrangement in thermal annealed film, our spherulites were characterized by both grazing-incident X-ray diffraction and X-ray diffraction data (Figures 2G, S24, and S25). As presented in Figures 2G and S25, a broad diffraction ring with Q ≈ 1.50 Å^−1^ along the out-of-plane and in-plane directions reveals the amorphous nature of DOSFX-SFXSO in the pristine spin-coated film. Interestingly, after thermal annealing, regular scattering patterns (Q_z_ = 0.26 Å^−1^ and 0.52 Å^−1^, 100 and 200) are observed in the out-of-plane direction, indicating a lamella-like orientation in our spherulites. The interlayer distance (d = 24.20 Å) is comparable to the calculated conjugated backbone length (approximately 25.0 Å) of DOSFX-SFXSO, as illustrated in Figure 2H. Other slightly pattern plots at Q_z_ = 0.58 Å^−1^ (10.8 Å, 002) are also observed, attributed to the interchain distance and consistent with the calculated side chain distance (10.5Å) of DOSFX-SFXSO. Besides, we also attribute the stronger pattern plot at 0.85 Å^−1^ (d = 7.38 Å, 010 and 020) to the intermolecular π-π interaction (Figure 2I). Therefore, it is easy to conclude that the molecular packing model is similar to the single crystal of DOSFX-SFXSO, but the molecules may have a large torsional angle of >165° in supramolecular spherulites. In this regard, we assumed that molecules show oriented and ordered planar conformation with a periodic and dense arrangement, which further self-assembles into higher level well-defined spherulites. In turn, we found out that our supramolecular framework is a key molecular design to induce planar conformation and obtain supramolecular spherulites.

### Excitonic Behavior of Steric Terfluorene-Based Supramolecular Spherulites

In light-emitting conjugated solid system, defects resulting from both physical and chemical irregularities, can induce nonemissive interchain polaron pairs, and exciton-exciton annihilation, leading to low emission efficiency and unstable emission behavior (Haedler et al., 2015, Podzorov, 2013, Spano and Silva, 2014, Xie et al., 2012). Beyond possible structural defects, well-ordered condensed structures will provide an “excitonic landscape channel” to suppress physical defect emission and to ensure high light radiation (Haedler et al., 2015, Podzorov, 2013). Compared with the amorphous state, the β-conformation in polyfluorene is found to be an energetically favorable environment for excitons, including singlet and triplet exciton, associated with their oriented conformation and long effective conjugation length (Ariu et al., 2003, Hayer et al., 2005; Prins et al., 2006). As discussed in the first section, shoulder absorption peaks at 407 nm for DOSFX-SFXSO annealed films clearly reveal the formation of planar conformation. In addition, the red-shifted and well-resolved PL spectra with a more vibrational structure further confirmed this assumption. The peaks of DOSFX-SFXSO β-conformation in the room temperature (RT) PL spectra are located at 425, 445, 472, and 513 nm. At 425 nm, the PL peak has been assigned to the S1ssi (0-0) transition. The β-conformation 0-0 PL emission peak is thus red-shifted by 20 nm compared with the 0-0 peak detected from the amorphous ones, attributable to the smaller energy gap for the β-conformational molecules (Lin et al., 2014, Liu et al., 2016, Yu et al., 2016). The intensity ratio of 0-0 to 0-1 emission band are high in spin-coated amorphous films, indicating an enhancement of the Huang-Rhys parameter and resulting from a larger configuration and relaxation in the excited states (Eggimann et al., 2019).

**Figure 3:**
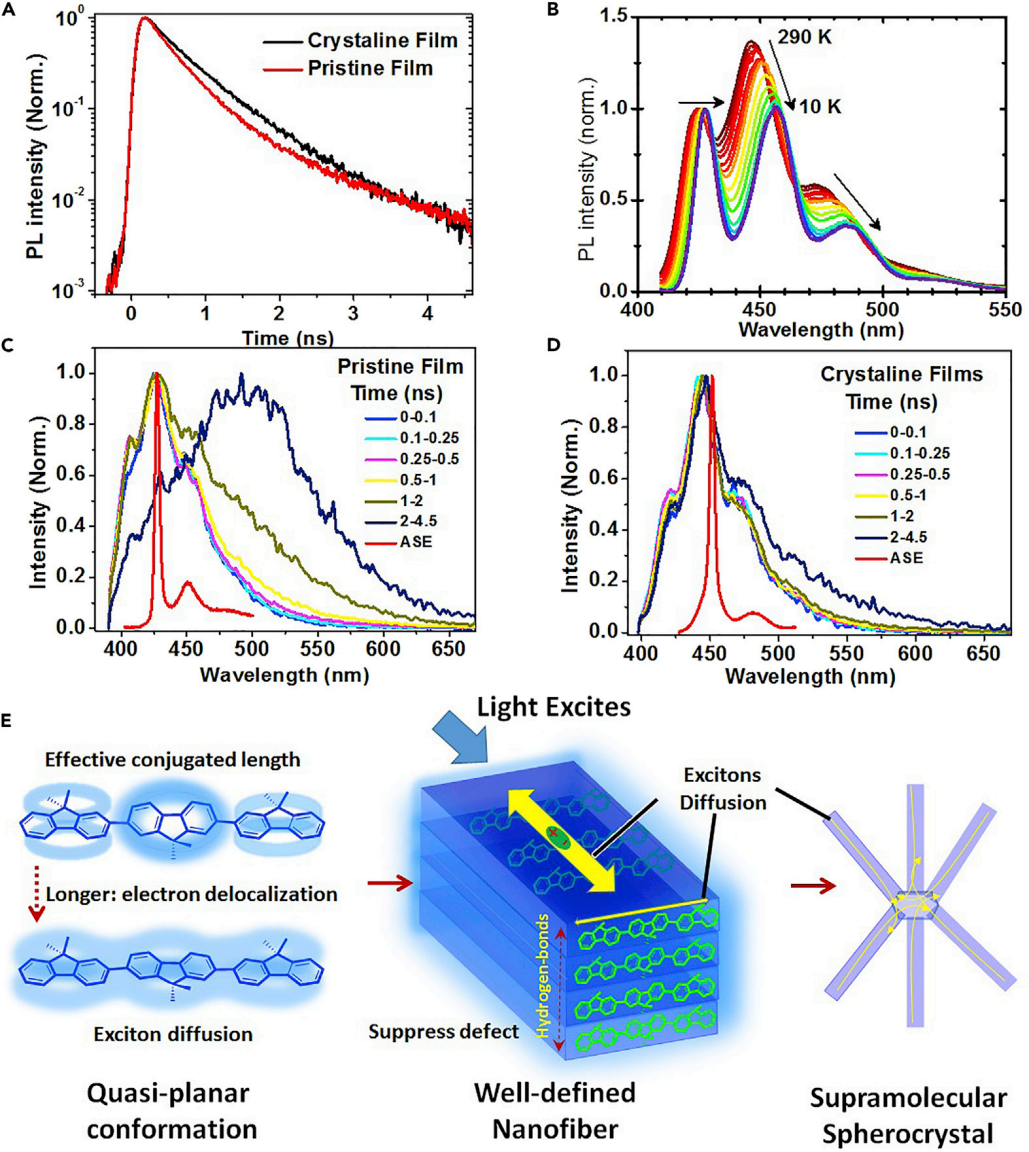
Transient Grating PL Spectra of DOSFX-SFXSO in Various States (A) Normalized PL kinetics of DOSFX-SFXSO and DOSFX-SFX amorphous spun films and β-conformation annealed films. (B–D) (B) Temperature-dependent PL spectra of DOSFX-SFXSO β-conformation annealed films from 10 K to 290 K. The PL spectra of DOSFX-SFXSO amorphous (C) and β-conformation (D) films excited at 365 nm using a frequency-doubled, mode-locked Ti:sapphire laser and measured at 0–4.5 ns following excitation. The ASE spectra of two DOSFX-SFXSO films were excited by 355 nm laser. (E) Schematic illustration of exciton diffusion in our well-defined condensed spherocrystals. Complete electron delocalization along p-orbit conjugated backbone structure enables quasi-planar conformational molecular structure to promote exciton diffusion.

As indicated in Figure 3A, the lifetime of pristine film and crystalline film are 380 and 530 ps, respectively, indicating that DOSFX-SFXSO β-conformation leads to the increase in PL lifetime, that crystalline phase is able to block defect emission. It can be seen that in the β-conformation PL spectra, emission peaks red-shift by 13 nm as the temperature is reduced from RT to 5 K (Figures 3B and S26), which might be the result of the reduced vibronic coupling of the fluorene units. The increase in the intensity of the 0-0 peak in PL spectra at low temperatures seems to suggest that the terfluorene chain is more planar, indicating that the contribution from increased π-electron delocalization plays a more significant role than assumed. The 0-1 vibronic PL peaks of crystalline-phase DOSFX-SFXSO do not split like PFO and PODPF β-conformation at lower temperatures. The reason we do not observe the vibrational fine structure is because the fraction of the material in the crystalline phase is much larger than that of PODPF/PFO in the β-conformation. This leads to an inhomogeneous broadening of the peaks. We see similar trends for PODPF β-conformation low-temperature PL spectra with a red-shift in emission, decreasing to 0-1 vibronic peak intensity, and increasing to 0-0/0-1 peak spacing with decreasing temperature.

According to previous work, serious fluorescence quenching usually occurs in a highly crystalline condensed structure of conjugated molecules, such as aggregation-induced quenching resulting to small radiative decay rate (k_r_) and high trapping rate (Jin et al., 2018). For example, the PL quantum efficiencies of DOSFX-SFX annealed films are approximately 16%, which is a decrease of nearly 70% from those of pristine spin-coated films (48%) (Table 1). Notably, photoluminescent quantum yield (PLQY) is 58% and 55% for DOSFX-SFXSO pristine and annealed films, and 45% and 36% for DHSFX-SFXSO ones, respectively, which are significantly different from those of conventional light-emitting conjugated molecules (Table 1). Increased degrees of structural order can reasonably suppress physical defects and enable crystalline structures to show robust emission. Besides, this assumption is also effectively confirmed by the well-resolved vibronic structure and narrow spectral profiles in PL decay from planar conformation and amorphous spin-coated films at RT following ultrafast excitation at 365 nm (Figures 3C and 3D). Figures 3C, 3D, and S27 show the time-resolved PL spectra of DOSFX-SFXSO and DSFX-OSFX films at three different times following 365-nm excitation. The most striking observation is that for all observations between 0 and 4.5 ns, the emission spectra from the β-conformation film are significantly different from those observed in the amorphous ones. As expected, in amorphous DOSFX-SFXSO and DSFX-OSFX crystalline films, long-wavelength and broad green-band “defect” emissions at 500 nm are found in the PL spectra at 1.0–4.5 ns, originating from the nonradiative intermolecular aggregation-induced excimer (Cacialli et al., 2002, Eggimann et al., 2019). As we discussed above, DSFX-OSFX also shows excellent crystalline structure in annealed films, but it also had a residual strong defect emission at long-wavelength emission (520 nm) after a delay of 2.5 ns (Figure S27), indicating that residual physical defect “excimer” may not completely suppress in conventional crystalline structure without order and orientation at molecular level. Remarkably, the annealed DOSFX-SFXSO leads to slight increase in PL lifetime and reduction in the long-wavelength defect emission, indicating that the DOSFX-SFXSO crystalline phase is able to suppress the “defect” emission effectively (Figure 3E). This is possibly due to the low energy of the DOSFX-SFXSO crystalline phase, as seen in absorption and PL, similar to the β-conformational domain in PFO and PODPF. Well-defined condensed structures, consisting of oriented planar conformation and highly ordered molecular packing, will effectively suppress the defect emission in our crystalline nanostructure (Figure 3E). What's more, amplified spontaneous emission (ASE) peaks of DOSFX-SFXSO pristine and annealed films estimated at 426 and 452 nm, attributed to 0-1 band emission, also effectively confirmed the planarization of conjugated backbone structure in supramolecular spherulites (Figures 3C and 3D). Of course, there is also a low fraction of longer wavelength in our DHSFX-SFXSO β-conformation films, attributing to the disordered domain in the film states. The strong electronic coupling in amorphous films, also called a physical defect, is detrimental to emission behavior in solid states (Cacialli et al., 2002). In the last several decades, green-band emission is a big obstacle to the induction of unstable and low emission efficiency, which is a key problem in the construction of a high-performance stable fluorene-based semiconductor. Our observation strongly confirms that the aggregation-caused excimer formation is an approach to induce this green emission as reported by Vacha et al. (Honmou et al., 2014, Nakamura et al., 2018).

**Table 1:** Photophsical Property of Selective Terfluorenes in Various States

Entry	λ_Abs max_ (nm)			λ_PL max_ (nm)			η			
	Sol.	Film	Ann.	Sol.	Film	Ann.	Sol.	Film	Ann	Enh.
DOSFX-SFX	354	359	360	397, 420	406, 427	405, 423, 452	96%	48%	16%	−67%
DOSFX-SFXSO	356	360	363, 382, 407	399, 420	410, 430	427, 450, 476	90%	58%	55%	−5%
DOF-SFXSO	356	360	359	400, 422	410, 430	407, 429, 453	89%	36%	20%	−45%
DOPhF-SFXSO	357	359	358	398,421	405, 426	404, 427, 452	92%	40%	20%	−50%
DHSFX-SFXSO	355	359	360, 378, 404	399, 420	410, 430	406, 427, 447, 475	95%	45%	36%	−20%
DSFX-OSFX	357	357	369, 390	395, 418	401, 423	403, 423, 452	98%	62%	38%	−40%

Sol., diluted solution; Ann., annealed films; Enh., increased η ratio of film states after thermal annealing.

In summary, we have designed and synthesized a range of terfluorenes with various side chain and substituted steric units. Interestingly, supramolecular DOSFX-SFXSO shows an unusually planar conformation (i-conformation) in the annealed film with perfect supramolecular spherulites resulting from the synergistic effects of hydrogen bonds and steric interaction. Strikingly, with regard to the crystalline-quenched emission in conventional light-emitting solid states, the strong luminance of our ordered and uniform artificial architectures is obtained with a high PLQY of 55%. In our assumption, this unique property is attributable to two factors: the β-conformation provides an energetically favorable environment for excitons. Meanwhile, the undesirable physical defects, such as aggregation-induced excimer, are effectively suppressed, as confirmed by the transient PL kinetics measurement. Long wavelength emission at 500 nm was observed in pristine films but absent in annealed films, which strongly supported the aggregation mechanism in fluorene green-band emission. In combination with the high molecular packing, such a crystalline-enhanced emission in our designed ordered artificial architectures could enable light-emitting optoelectronic devices that apply this well-defined structure as a photoelectric conversion antenna. These could contribute to the construction of high-performance organic light-emitting field transistors and electrical pumped organic lasers.

### Limitations of the Study

In this study, the DOSFX-SFXSO with planar conformation in the annealed film can easily self-organize into perfect supramolecular spherulites via the synergistic effects of hydrogen bonds and steric interaction. However, accurate roles of hydrogen bondings and molecular packing behaviors in conformational planarization are not fully understood here, and electronic properties of spherulites and microcrystals need to be further investigated.

## Methods

All methods can be found in the accompanying Transparent Methods supplemental file.

## Supplemental Information

Document S1. Transparent Methods, Figures S1–S49, Schemes S1 and S2, and Tables S1 and S2

Data S1. CIF file of DHSFX-SFXSO, Related to Figure 2

Data S2. CIF file of DOF-SFXSO, Related to Figure 2

Data S3. CIF file of DOSFX-SFX, Related to Figure 2

Data S4. CIF file of DOSFX-SFXSO, Related to Figure 2
