# Supplementary material for: Hierarchical Uniform Supramolecular Conjugated Spherulites with Suppression of Defect Emission
Source: iScience. 2019 Jun 8;16:399–409. doi: 10.1016/j.isci.2019.06.002 (PMC6593144; doi:10.1016/j.isci.2019.06.002)
Supplement: Document S1. Transparent Methods, Figures S1–S49, Schemes S1 and S2, and Tables S1 and S2 [file mmc1.pdf]

ISCI, Volume *16*

Supplemental Information

Hierarchical Uniform Supramolecular Conjugated Spherulites with Suppression of Defect Emission

Changjin Ou, Nathan J. Cheetham, Jiena Weng, Mengna Yu, Jinyi Lin, Xuhua Wang, Chen Sun, Juan Cabanillas-Gonzalez, Linghai Xie, Lubing Bai, Yamin Han, Donal D.C. Bradley, and Wei Huang

Supplemental Information

Transparent Methods

Materials and reagents

All reagents were purchased from Sigma-Aldrich, Merck and Alfa Aesar, and used without further purification unless stated otherwise. Anhydrous THF was dried by sodium using benzophenone as indicator. The synthetic routes of spiro[fluorene-9,9'-thioxanthene] 10',10'-dioxide and 2-bromo-3',6'-bis(octyloxy)spiro[fluorene-9,9'-xanthene] were followed the literatures (Ou and Zhu, et al., 2017; Ou and Ding, et al., 2017; Zuo, et al., 2018).

Characterization

^{1}H and ^{13}C NMR spectrum was recorded on a Bruker 400 MHz spectrometer in $CDCl_3$ with tetramethylsilane (TMS) as the interval standard. Absorption spectra were measured with a Shimadzu UV-3600 spectrometer at 25 ^{o}C, and emission spectra were recorded on a Shimadzu RF-5301(PC) luminescence spectrometer. DSC measurement was acquired using a Shimadzu Instruments DSC-60A. DSC data were collected at a rate of 10 ^{o}C/min for both of the baseline and sample. Thermogravimetric analyses (TGA) were conducted by a Shimadzu DTG-60H under a heating rate of 10 ^{o}C/min and a nitrogen flow rate of 50 cm^{3}/min. Cyclic voltammetric (CV) studies were conducted using an CHI660C Electrochemical Work station in a typical three-electrode cell with a platinum sheet working electrode, a platinum wire counter electrode, and a silver/silver nitrate (Ag/Ag^{+}) reference electrode. All electrochemical experiments were carried out under a nitrogen

atmosphere at room temperature in an electrolyte solution of 0.1 M tetrabutylammonium hexafluorophosphate (n-Bu_4NPF_6) in CH_2Cl_2 at a sweeping rate of 0.1 V/s. According to the redox onset potentials of the CV measurements, the HOMO/LUMO energy levels of the materials are estimated based on the reference energy level of ferrocene (4.8 eV below the vacuum): HOMO/ LUMO = $-[E_{onset} - E_{(Fc/Fc+)} + 4.8]$ eV. For scanning electron microscopic (SEM) studies, a drop of 20 μL solutions were placed onto silicon substrates, and the solvent was left to evaporate. The samples were then examined with a field emission SEM (Hitachi, S-4800) at an accelerating voltage of 3 kV. The film morphologies of films were recorded with a Bruker's Dimension Icon AFM in tapping mode (Bruker's Sb/Si probe tip with a resonant frequency 320 kHz and the spring constant 42 Nm^{-1}). The Single crystal data collection was performed at 100 or 298 K on a Bruker 2000 CCD area detector using graphite-monochromated Mo Kα radiation (λ = 0.71073 Å). All structures were solved by direct methods using OLEX2 and refined against F^2 using SHELXL-2014. Hydrogen atoms were fixed geometrically and refined isotropically. To measure the fluorescence lifetime, the incident 390 nm, 150-fs laser pulses were generated from a Coherent TOPAS-C optical parametric amplifier; pumped by a 1 kHz Coherent Legend regenerative amplifier that is seeded by a Coherent Vitesse oscillator. These input laser pulses were focused by a lens (f = 20 cm) on the samples solution in a 1-mm-thick quartz cell (beam spot ~1 mm inside the cell). The emission from the samples was collected at a backscattering angle of 150° by a pair of lenses and directed to an Optronis Optoscope™ streak camera system which has an ultimate

temporal resolution of 6 ps. CD spectra were collected using a JASCO J-820 spectropolarimeter (Tokyo, Japan) and CD measurements were from 300 to 450 nm, the data pitch was 0.5 nm, scan speed was 800 nm/min, response time was 0.5 s, and bandwidth was 1 nm. GIXD measurements were performed on Beamline 7.3.3 at the Advanced Light Source (ALS) at the Lawrence Berkeley National Laboratory. An X-ray beam impinged onto the sample at a grazing angle above and below the critical angle of the polymer film (αc = 0.16), but below the critical angle of the silicon substrate (αc = 0.22). The wavelength of X-rays was 1.240 Å, and the scattered intensity was detected by PILATUS 1M detector. The sample was loaded on silicon nitride substrate, and the beam energy used here is 284.4 eV which generally probes the carbon edge resonance.

ASE characterization

For ASE characterization, second harmonic generation (400 nm) of a re-generative amplifier (Spitfire, spectra Physics) was used as the optical pump source. The spot size of the pump laser beam focused on the samples was about 0.13 mm in diameter, which was perpendicular to the sample, and pump energy was controlled by an attenuator wheel and measured by laser power meter. The emission was detected by the optical fiber and then dispersed to a monochromator equipped with a charge coupled device detector (iDus, Andor).

Scheme S1. The synthetic route of 2,7-bis(4,4,5,5-tetramethyl-1,3,2-dioxaborolan-2-yl)spiro[fluorene-9,9'-thioxanthene] 10',10'-dioxide.

~~Synthesis procedures~~

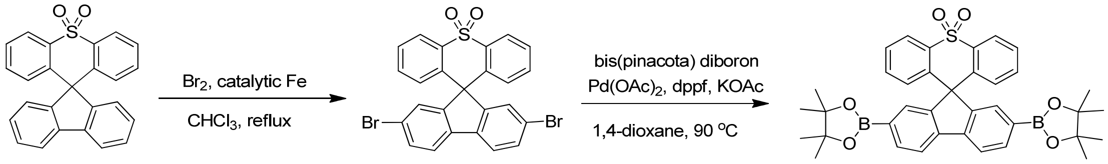

***Scheme* S2. The synthetic routes of bulk terfluorenes.**

~~Scheme S1. The synthetic route of 2,7-bis(4,4,5,5-tetramethyl-1,3,2-dioxaborolan-2-yl)spiro[fluorene-9,9'-thioxanthene] 10',10'-dioxide.~~

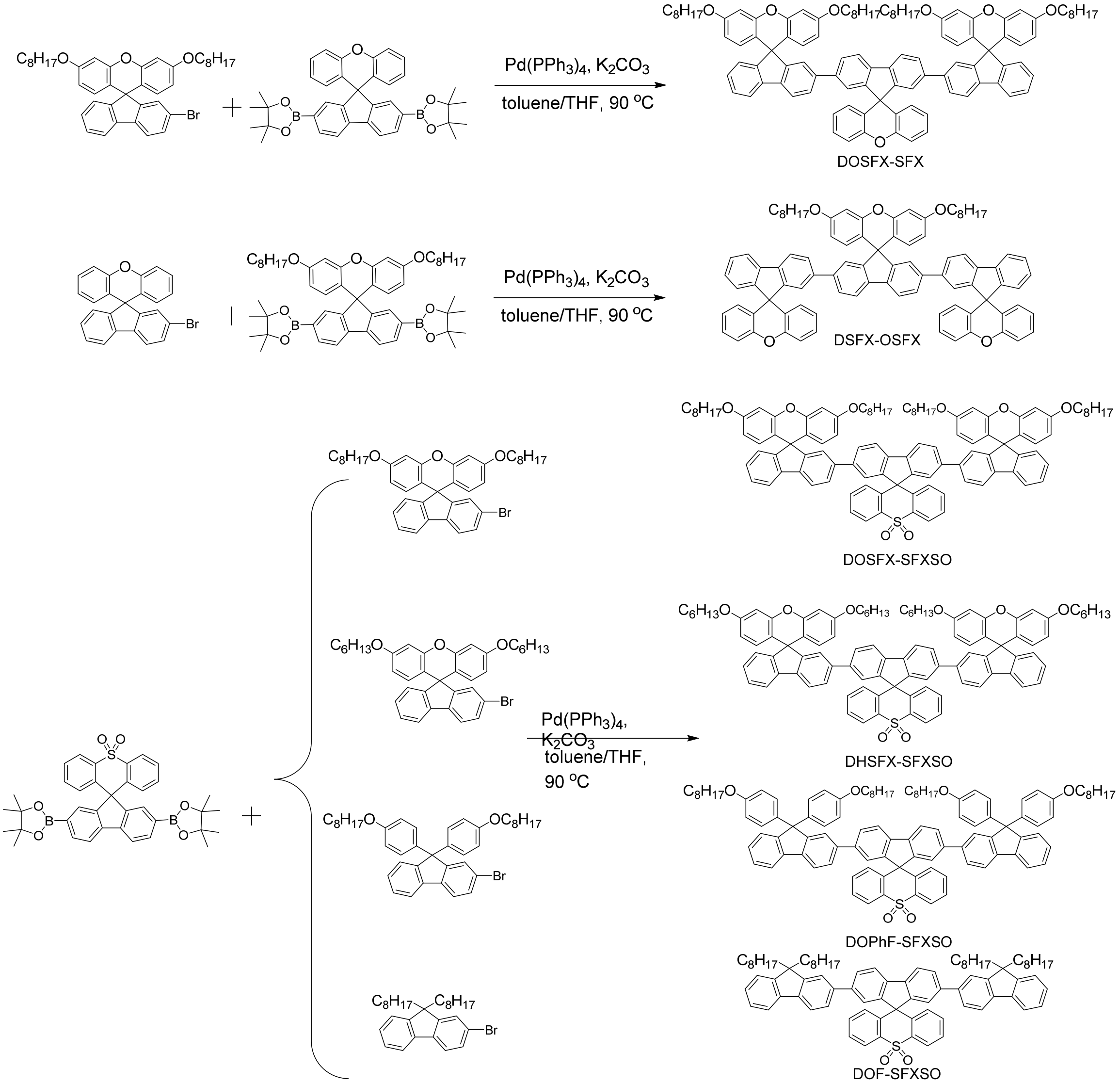

***~~Scheme~~* ~~S2. The synthetic routes of bulk terfluorenes.~~**

Synthesis of 2,7-dibromospiro[fluorene-9,9'-thioxanthene] 10',10'-dioxide

To a 2-kneck round flask, spiro[fluorene-9,9'-thioxanthene] 10',10'-dioxide (1.9 g, 5 mmol) and catalytic amount Fe powder was added, and then dissolved with chloroform (50 mL). Bromine (1.84 g, 11.5 mmol) was added, and then heated to reflux overnight. Sodium hydrogen sulfite aqueous solution was added to quench the excess of bromine, and then extracted with dichloromethane. The combined organic abstracted was dried over anhydrous $MgSO_4$ and filtered. The solution was evaporated under vacuum condition. The product was purified with silica gel column chromatography to afford a white solid (2.5 g, 92.6%). ^{1}H NMR(400 Mz, $CDCl_3$) δ 8.26 (dd, J = 8.0, 1.2 Hz, 2H), 7.69 (d, J = 8.0 Hz, 2H), 7.57 (dd, J = 8.0, 1.6 Hz, 2H), 7.52 (t, J = 7.6 Hz, 2H), 7.46 (d, J = 1.6 Hz, 2H), 7.32 (td, J = 8.8 Hz, 2H), 6.56 (d, J = 8.0 Hz, 2H). ^{13}C NMR(100 Mz, $CDCl_3$) δ 153.81, 138.42, 138.28, 136.79, 133.07, 132.20, 129.13, 128.91, 128.79, 123.71, 123.01, 121.79, 57.75.

Synthesis of 2,7-bis(4,4,5,5-tetramethyl-1,3,2-dioxaborolan-2-yl)spiro [fluorene-9,9'-thioxanthene] 10',10'-dioxide.

Under N_2 atmosphere, a mixture of 2,7-dibromospiro[fluorene-9,9'-thioxanthene] 10',10'-dioxide (2.69 g, 5 mmol), bis(pinacolato)diboron (3.04 g, 12 mmol), $Pd(OAc)_2$ (0.11 g, 0.5 mmol), dppf (0.55 g, 1 mmol), KOAc (1.47 g, 15 mmol) and 1.4-dioxahaxane (50 mL) was added into a 2-kneck round flask, then stirred and heated 90 ^{o}C for 6 hours. The reaction mixture was poured into water and extracted with ethyl acetate. The combined extracts were dried by anhydrous $MgSO_4$, and then the solvent was evaporated under vacuum condition. The crude product was purified by silica gel column chromatography to afford with solid (2.92 g) with 92.4% yield.

^{1}H NMR (400 Mz, $CDCl_3$) δ 8.22 (dd, J = 8.0, 1.2 Hz, 2H), 7.88 (s, 4H), 7.62 (s, 2H), 7.44 (t, J = 7.6 Hz, 2H), 7.21 (dt, J = 7.6, 1.6 Hz, 2H), 6.50 (d, J = 7.6Hz, 2H), 1.26 (s, 24H). ^{13}C NMR (100 Mz, $CDCl_3$) δ 152.83, 143.03, 139.21, 136.69, 135.23, 132.66, 131.78, 129.41, 128.20, 123.32, 120.07, 83.96, 57.61, 24.82.

Synthesis of DOSFX-SFX

Under N_2 atmosphere, a mixture of 2,7-di(1,3,2-dioxaborolan-2-yl) spiro[fluorene-9,9'-xanthene] (0.47 g，1.0 mmol), 2-bromo-3',6'-bis(octyloxy)spiro [fluorene-9,9'-xanthene] (1.77 g，2.2 mmol), $Pd(PPh_3)_4$ (0.115 g, 0.1 mmol), 2M K_2CO_3 solution (2.0 ml) and toluene/THF (20 ml, 1:1 v/v) was stirred and heated 90°C for 24h. The mixture was abstracted with dichloromethane, and then the combined abstracts were dried by anhydrous $MgSO_4$. The solvent was evaporated under vacuum condition. The crude product was purified by silica gel column chromatography to afford white solid (0.98 g) with 65.1% yield. ^{1}H NMR(400 Mz, $CDCl_3$) δ 7.74-7.71(m, 6H), 7.47(dd, J = 8.0, 1.6 Hz, 2H), 7.43(dd, J = 8.0, 1.6 Hz, 2H), 7.31(t, J = 7.6 Hz, 2H), 7.29(d, J = 4.0 Hz, 4H), 7.23(dd, J = 8.0, 1.6 Hz, 2H), 7.19(dd, J = 7.6 Hz, 2H), 7.17-7.15(m, 2H), 7.09(d, J = 7.6 Hz, 2H), 6.76(dd, J = 7.6, 1.6 Hz, 2H), 6.71(d, J = 1.6 Hz, 4H), 6.44(dd, J = 8.0, 2.4 Hz, 2H), 6.33(dd, J = 8.4, 2.4 Hz, 4H), 6.27(d, J = 8.4 Hz, 4H), 3.91(t, J = 6.8 Hz, 8H), 1.78-1.71(m, 8H), 1.46-1.39(m, 8H), 1.34-1.27(m, 32H), 0.89(t, J = 2.8 Hz, 12H). ^{13}C NMR (100 MHz, $CDCl_3$) δ 158.95, 156.11, 156.06, 155.93, 152.03, 151.31, 141.25, 141.09, 139.14, 138.79, 138.44, 128.79, 128.30, 128.19, 128.11, 127.57, 127.20, 126.96, 125.52, 124.71, 123.10, 124.08, 123.41, 120.10, 119.97, 119.85, 116.82, 116.51, 111.02,

101.68, 68.12, 54.38, 53.48, 31.82, 29.34, 29.23, 29.21, 26.06, 22.66, 14.10.

Synthesis of DSFX-OSFX

Under N_2 atmosphere, a mixture of 2-bromospiro[fluorene-9,9'-xanthene] (1.23 g，3.0 mmol), 2,2'-(3',6'-bis(octyloxy)spiro[fluorene-9,9'-xanthene]-2,7-diyl)bis(4,4,5,5-tetramethyl-1,3,2-dioxaborolane) (0.84 g，1.0 mmol), $Pd(PPh_3)_4$ (0.115 g, 0.1 mmol), 2M K_2CO_3 solution (2.5 ml) and toluene/THF (30 ml, 1:1 v/v) was stirred and heated 90 ^oC for 18h. The mixture was abstracted with dichloromethane, and then the combined abstracts were dried by anhydrous $MgSO_4$. The solvent was evaporated under vacuum condition. The crude product was purified by silica gel column chromatography to afford white solid (0.93 g) with 74.4% yield. ^1H NMR (400 MHz, $CDCl_3$) δ 7.76 (d, J = 8.0 Hz, 2H), 7.74 (d, J = 8.0 Hz, 2H), 6.69 (d, J = 8.0 Hz, 2H), 7.47 (d, J = 8.0 Hz, 2H), 7.43 (d, J = 8.0 Hz, 2H), 7.33 (t, J = 7.6 Hz, 2H), 7.32 (s, 2H), 7.25 (s, 2H), 7.21 (d, J = 8.0 Hz, 4H), 7.19-7.12 (m, 8H), 6.76 (td, J = 7.6, 1.2 Hz, 2H), 6.70 (d, J = 2.0, 2H), 6.42 (d, J = 7.6, 4H), 6.32 (dd, J = 8.8, 2.0 Hz, 2H), 6.28 (d, J = 8.8 Hz, 2H), 3.91 (t, J = 6.8, 4H), 1.77-1.71 (m, 4H), 1.44-1.38 (m, 4H), 1.31-1.25 (m, 16H), 0.89 (t, J = 6.8 Hz, 6H). ^{13}C NMR (100 MHz, $CDCl_3$) δ 158.94, 156.55, 155.71, 155.31, 151.98, 151.35, 141.28, 141.08, 139.27, 138.81, 138.38, 128.92, 128.32, 128.08, 128.03, 127.76, 127.20, 126.96, 125.58, 124.81, 124.18, 124.00, 123.33, 120.04, 120.00, 119.95, 116.75, 116.38, 111.04, 101.71, 68.12, 54.31, 53.56, 31.81, 29.70, 29.33, 29.22, 26.05, 22.65, 14.09.

Synthesis of DOSFX-SFXSO

Under N_2 atmosphere, a mixture of 2-bromo-3',6'-bis(octyloxy)spiro[fluorene-9,9

'-xanthene] (1.34 g, 2.0 mmol), 2,7-bis(4,4,5,5-tetramethyl-1,3,2-dioxaborolan-2-yl) spiro[fluorene-9,9'-thioxanthene] 10',10'-dioxide (0.59 g, 0.93 mmol), $Pd(PPh_3)_4$ (0.107 g, 0.092 mmol), 2M K_2CO_3 solution (1.8 ml) and toluene/THF (20 ml, 1:1 v/v) was stirred and heated 90°C for 24h. The mixture was abstracted with dichloromethane, and then the combined abstracts were dried by anhydrous $MgSO_4$. The solvent was evaporated under vacuum condition. The crude product was purified by silica gel column chromatography to afford white solid (0.89 g) with 61.7% yield. ^{1}H NMR(400 Mz, $CDCl_3$) δ 8.25(dd, J = 8.0, 1.2 Hz, 2H), 7.74(t, J = 8.0 Hz, 4H), 7.72(d, J = 8.0 Hz, 2H), 7.56(s, 2H), 7.52(dd, J = 8.0, 1.6 Hz, 2H), 7.46(d, J = 8.0 Hz, 4H), 7.31(t, J = 7.6 Hz, 2H), 7.28(s, 2H), 7.24(td, J = 8.0, 1.2 Hz, 2H), 7.17(t, J = 7.6 Hz, 2H), 7.08(d, J = 7.6 Hz, 2H), 6.70(sd, J = 2.4 Hz, 4H), 6.62(d, J = 7.6 Hz, 2H), 6.33(d, J = 8.4, 2.4 Hz, 4H), 6.26(d, J = 8.4 Hz, 4H), 3.90(t, J = 6.4 Hz, 8H), 1.78-1.71(m, 8H), 1.45-1.38(m, 8H), 1.34-1.27(m, 32H), 0.88(t, J = 6.8 Hz, 12H). ^{13}C NMR(100 Mz, $CDCl_3$) δ 158.96, 156.07, 156.03, 153.08, 152.02, 141.62, 140.34, 139.96, 139.35, 139.16, 138.80, 136.78, 136.77, 132.98, 129.02, 128.83, 128.38, 127.64, 127.62, 126.80, 125.53, 124.06, 123.81, 123.45, 120.54, 120.28, 119.93, 116.45, 110.99, 101.66, 68.13, 58.18, 53.46, 31.82, 29.34, 29.23, 29.21, 26.06, 22.66, 14.11.

Synthesis of DHSFX-SFXSO

Under N_2 atmosphere, a mixture of 2-bromo-3',6'-bis(hexyloxy) spiro[fluorene-9,9'-xanthene] (1.22 g, 2.0 mmol), 2,7-bis(4,4,5,5-tetramethyl-1,3,2 -dioxaborolan-2-yl)spiro[fluorene-9,9'-thioxanthene] 10',10'-dioxide (0.59 g, 0.93

mmol), $Pd(PPh_3)_4$ (0.107 g, 0.092 mmol), 2M K_2CO_3 solution (1.9 ml) and toluene/THF (20 ml, 1:1 v/v) was stirred and heated 90°C for 24h. The mixture was abstracted with dichloromethane, and then the combined abstracts were dried by anhydrous $MgSO_4$. The solvent was evaporated under vacuum condition. The crude product was purified by silica gel column chromatography to afford white solid (0.94 g) with 70.1 % yield. ^{1}H NMR(400 Mz, $CDCl_3$) δ 8.25 (dd, J = 8.0, 1.2 Hz, 2H), 7.74 (t, J = 8.0 Hz, 4H), 7.72 (d, J = 8.0 Hz, 2H), 7.57 (s, 2H), 7.52 (dd, J = 8.0, 1.6 Hz, 2H), 7.46 (t, J =7.6 Hz, 4H), 7.32 (t, J = 7.6 Hz, 2H), 7.29 (s, 2H), 7.24 (td, J = 8.0, 1.2 Hz, 2H), 7.17 (t, J = 7.6 Hz, 2H), 7.09 (d, J = 7.6 Hz, 2H), 6.70 (sd, J = 2.4 Hz, 4H), 6.63 (d, J = 8.1 Hz, 2H), 6.33 (dd, J = 8.4, 2.4 Hz, 4H), 6.27 (d, J = 8.4 Hz, 4H), 3.91 (t, J = 6.4 Hz, 8H), 1.78-1.71(m, 8H), 1.46-1.39 (m, 8H), 1.33-1.29 (m, 16H), 0.89 (t, J = 6.8 Hz, 12H). ^{13}C NMR(100 Mz, $CDCl_3$) δ 158.95, 156.07, 156.03, 153.07, 152.01, 141.62, 140.34, 139.96, 139.34, 139.16, 138.80, 136.75, 133.00, 129.02, 128.83, 128.39, 127.64, 127.62, 126.80, 125.53, 124.06, 123.82, 123.45, 120.55, 120.28, 119.94, 116.44, 110.98, 101.65, 68.12, 58.17, 53.44, 31.57, 29.18, 25.73, 22.61, 14.05.

Synthesis of DOF-SFXSO

Under N_2 atmosphere, a mixture of 2-bromo-9,9-dioctylfluorene (1.17 g，2.5 mmol), 2,7-bis(4,4,5,5-tetramethyl-1,3,2-dioxaborolan-2-yl)spiro[fluorene-9,9'-thioxanthene] (0.63 g，1.0 mmol), $Pd(PPh_3)_4$ (0.12 g, 0.1 mmol), 2M K_2CO_3 solution (1.8 ml) and toluene/THF (20 ml, 1:1 v/v) was stirred and heated 90°C for 24h. The mixture was abstracted with dichloromethane, and then the combined abstracts were dried by

anhydrous $MgSO_4$. The solvent was evaporated under vacuum condition. The crude product was purified by silica gel column chromatography to afford white solid (0.95 g) with 82.1% yield. ^{1}H NMR(400 Mz, $CDCl_3$) δ 8.31(dd, J = 8.0, 0.8 Hz, 2H), 7.96 (d, J = 8.0 Hz, 2H), 7.81(dd, J = 8.0, 1.6 Hz, 2H), 7.77(s, 2H), 7.69-7.66 (m, 4H), 7.54-7.45 (m, 6H), 7.35-7.28 (m, 8H), 6.80 (d, J = 8.0 Hz, 2H), 1.96 (t, J = 8.0 Hz, 8H), 1.20-.1.13 (m, 8H), 1.12-1.02 (m, 32H), 0.80 (t, J = 6.8 Hz, 12H), 0.64-0.56 (m, 8H). ^{13}C NMR(100 Mz, $CDCl_3$) δ 153.22, 151.54, 151.02, 142.12, 140.81, 140.59, 140.16, 139.17, 138.75, 137.03, 132.95, 129.03, 128.47, 127.46, 127.09, 126.75, 125.75, 124.13, 123.59, 122.90, 120.91, 120.69, 120.00, 119.78, 58.36, 55.18, 40.31, 31.76, 29.99, 29.19, 23.76, 22.58, 14.07.

Synthesis of DOPhF-SFXSO.

Under N_2 atmosphere, a mixture of 2-(9,9-bis(4-(octyloxy)phenyl)fluoren-2-yl)-4,4,5,5-tetramethyl-1,3,2-dioxaboroane (1.75 g, 2.5 mmol), 2,7-dibromospiro[fluorene-9,9-thiaxanthene]-S,S-dioxide (0.54 g, 1.0 mmol), $Pd(PPh_3)_4$ (0.115 g, 0.1 mmol), 2M K_2CO_3 solution (2.0 ml) and toluene/THF (20 ml, 1:1 v/v) was stirred and heated 90°C for 24h. The mixture was abstracted with dichloromethane, and then the combined abstracts were dried by anhydrous $MgSO_4$. The solvent was evaporated under vacuum condition. The crude product was purified by silica gel column chromatography to afford white solid (1.25 g) with 81.9% yield. ^{1}H NMR(400 Mz, $CDCl_3$) δ 8.27(d, J = 8.0 Hz, 2H), 7.85(d, J = 8.4 Hz, 2H), 7.71(t, J = 7.6 Hz, 4H), 7.65(d, J = 2.4 Hz, 4H), 7.56(s, 2H), 7.50-7.45(m, 4H), 7.35-7.23(m, 8H), 7.11(d, J = 8.8 Hz, 8H), 6.73(d, J = 8.8 Hz, 8H), 6.69(d, J =

8.4 Hz, 2H), 3.88(t, *J* = 6.8 Hz, 8H), 1.77-1.70(m, 8H), 1.45-1.38(m, 8H), 1.35-1.28(m, 32H), 0.88(t, *J* = 6.8 Hz, 12H). ^{13}C NMR (100 Mz, $CDCl_3$) δ 157.89, 153.16, 152.67, 152.24, 141.88, 140.04, 139.64, 139.58, 139.40, 139. 19, 137.69, 136.94, 132.91, 129.17, 129.04, 128.40, 127.73, 127.60, 127.33, 126.38, 126.06, 124.22, 124.18, 123.53, 120.61, 120.47, 120.24, 114.10, 67.88, 64.26, 58.28, 31.83, 29.37, 29.32, 29.25, 26.10, 22.67, 14.12.

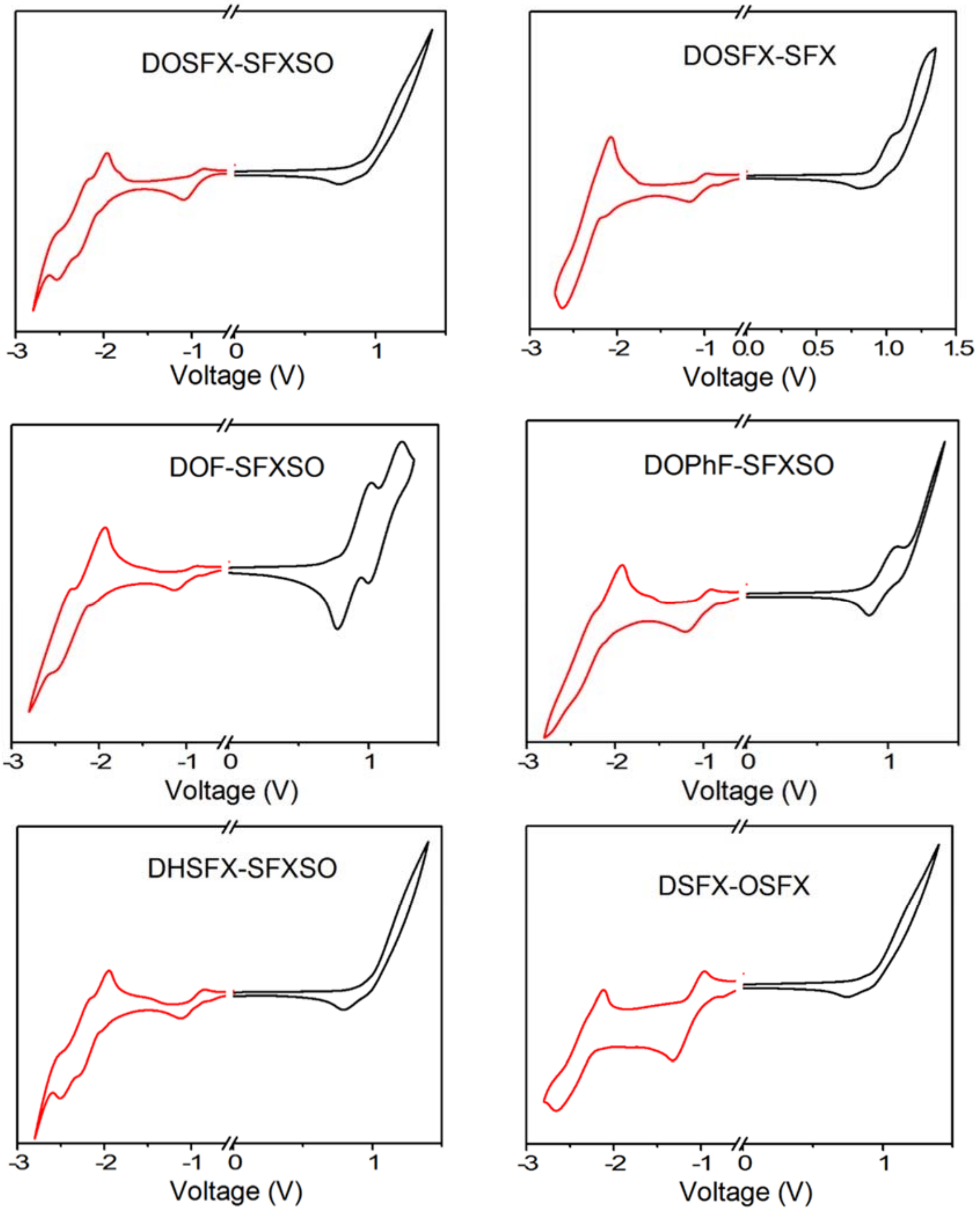

Figure S1. Cyclic voltammetry curves of terfluorene derivatives. The peak around -1.0 V is the reduction peak of water. Related to Figure 1.

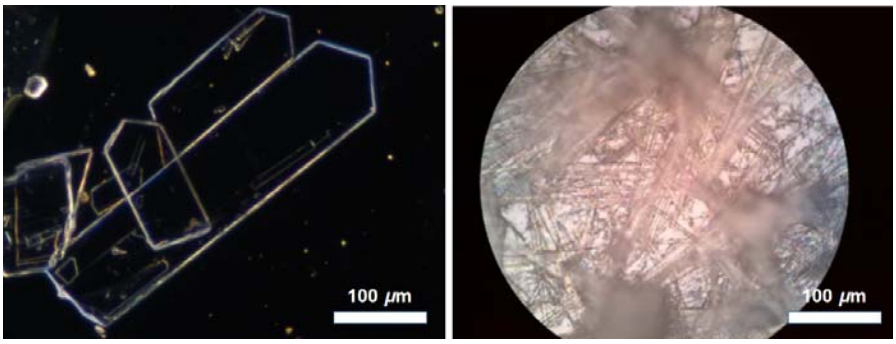

Figure S2. Optical microscope images of plate and needle crystals of DOSFX-SFXSO, the plate and needle crystals were obtained from ethanol-chloroform and methanol-dichloromethane solutions, respectively, the needle crystals were too tiny to perform single-crystal XRD. Related to Figure 2.

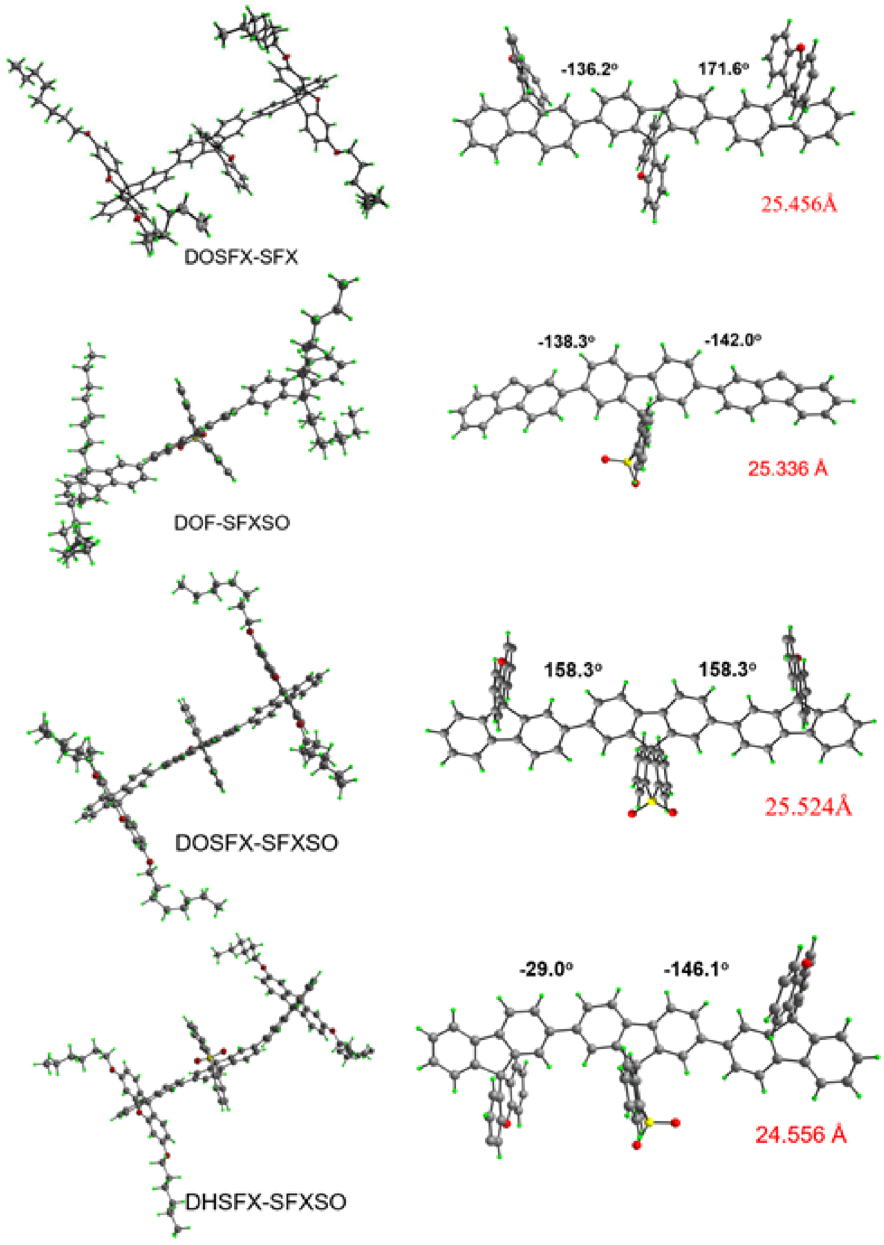

Figure S3. Crystal structures, torsional angles, and molecular length of conjugated backbone of spiro-terfluorenes. The alkyl or alkoxyl chains were omitted for clarity. Both DOSFX-SFX and DOF-SFXSO were crystallized from dichloromethane/ethanol solution, and the single crystals of DOSFX-SFXSO and DHSFX-SFXSO were obtained from chloroform/methanol solution, and contain chloroform and methanol molecules in lattice, respectively. In addition, both DOSFX-SFXSO and DHSFX-SFXSO can crystallize into needle crystals in dichloromethane/ethanol solution, while the crystals are too small to perform single X-ray diffraction. Related to Figure 2.

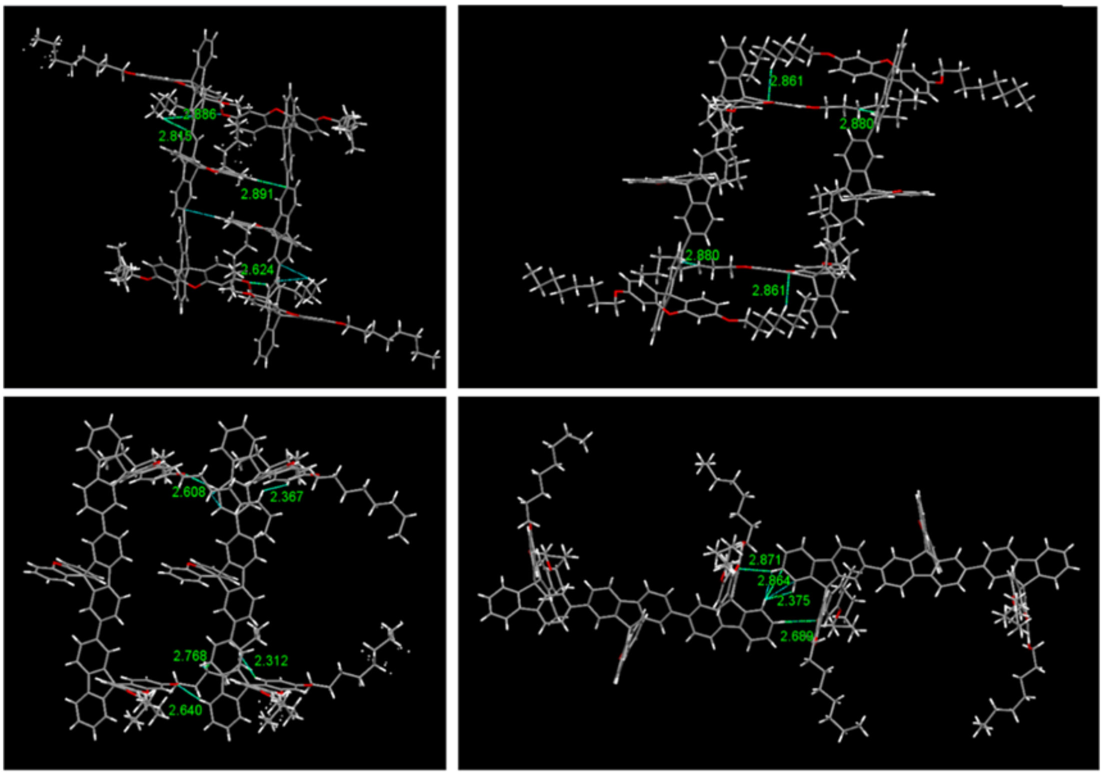

Figure S4. Intermolecular interactions between DOSFX-SFX dimers. Related to Figure 2.

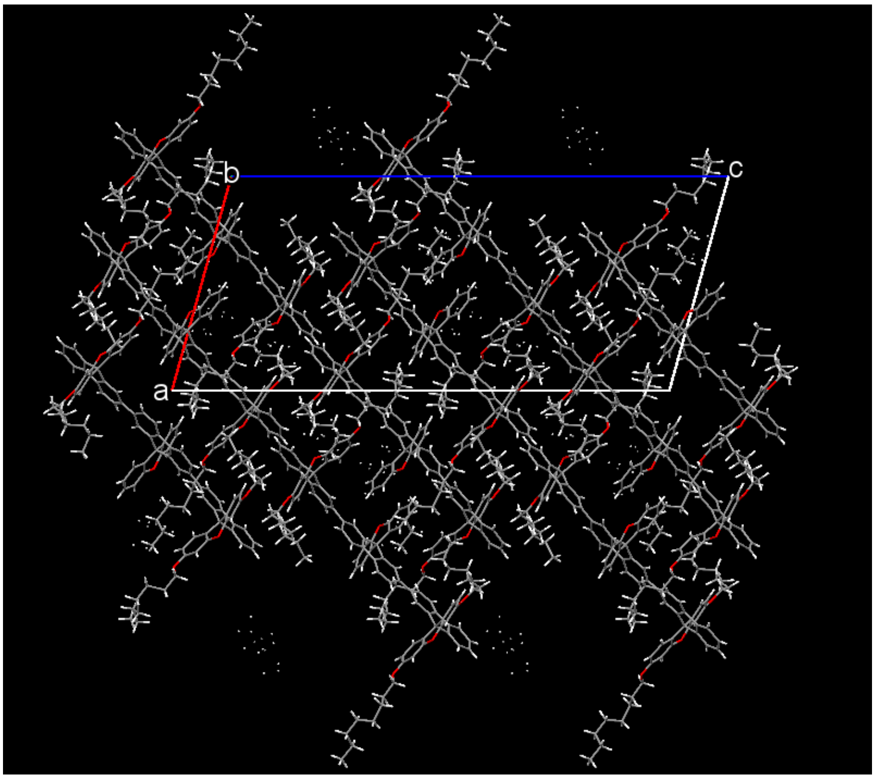

Figure S5. Packing motif of DOSFX-SFX, view from b-axis. Related to Figure 2.

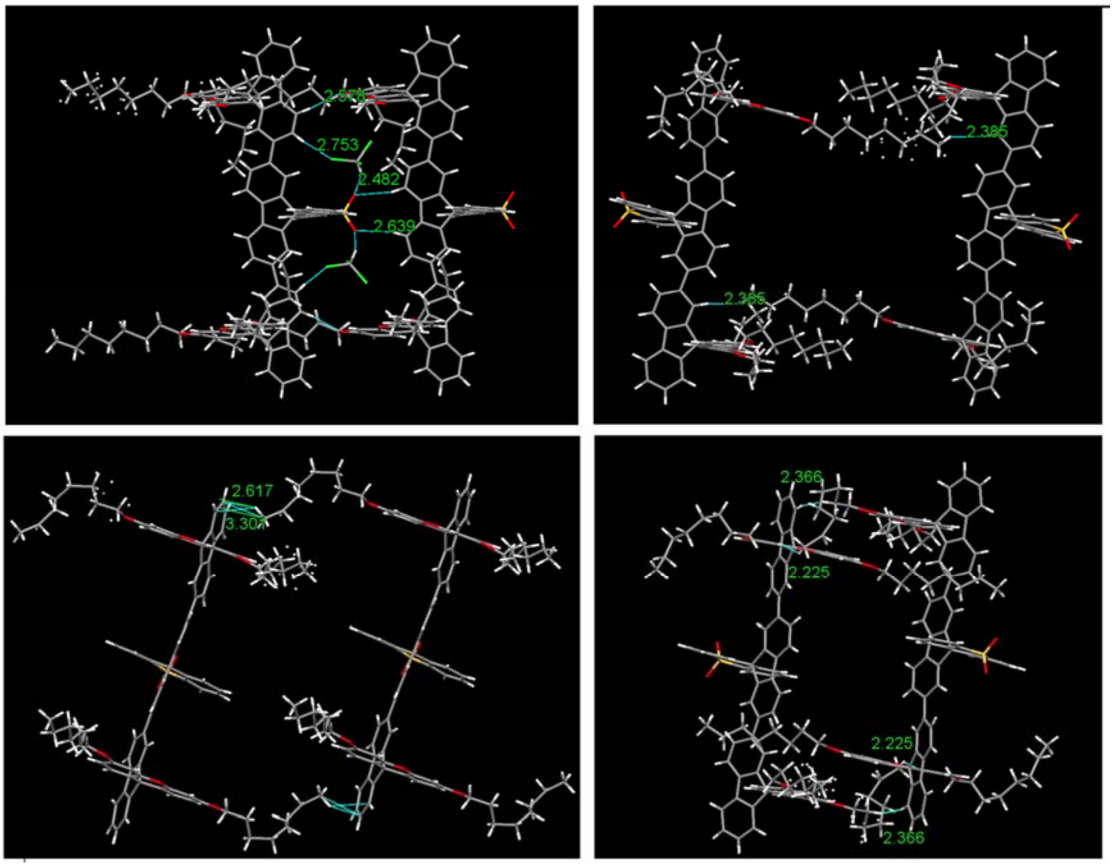

Figure S6. Intermolecular interactions in DOSFX-SFSO crystal. Related to Figure 2.

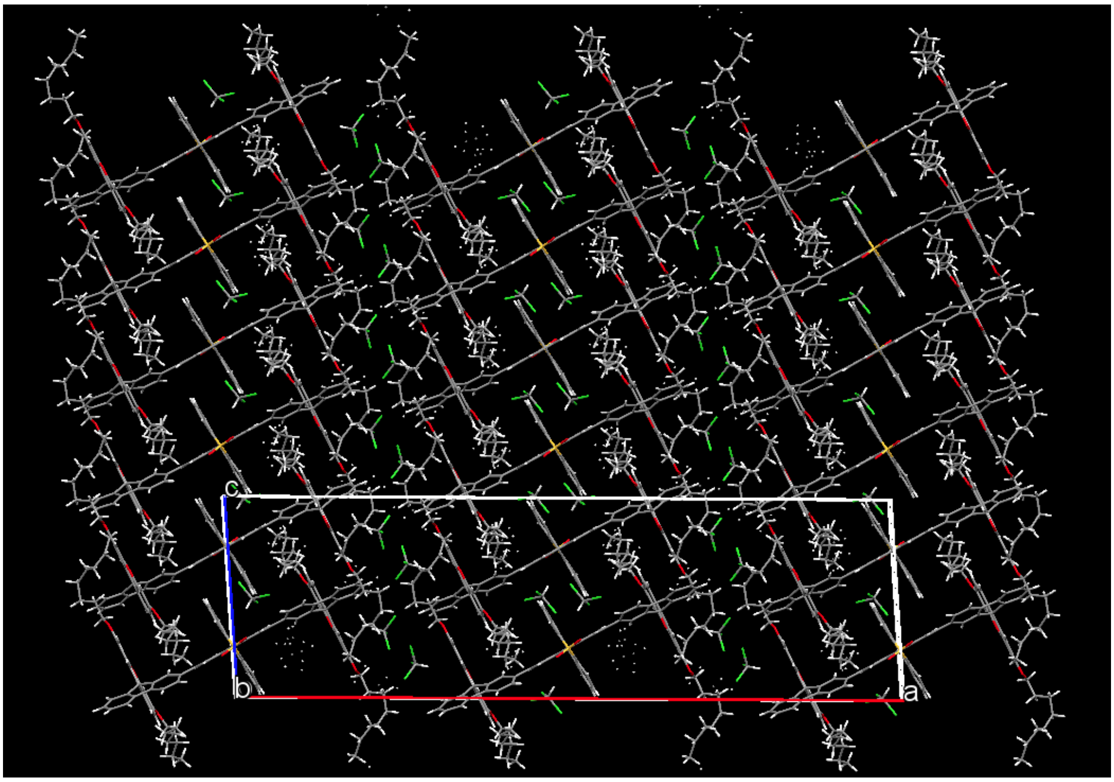

Figure S7. Packing mode of DOSFX-SFSO crystal. Related to Figure 2.

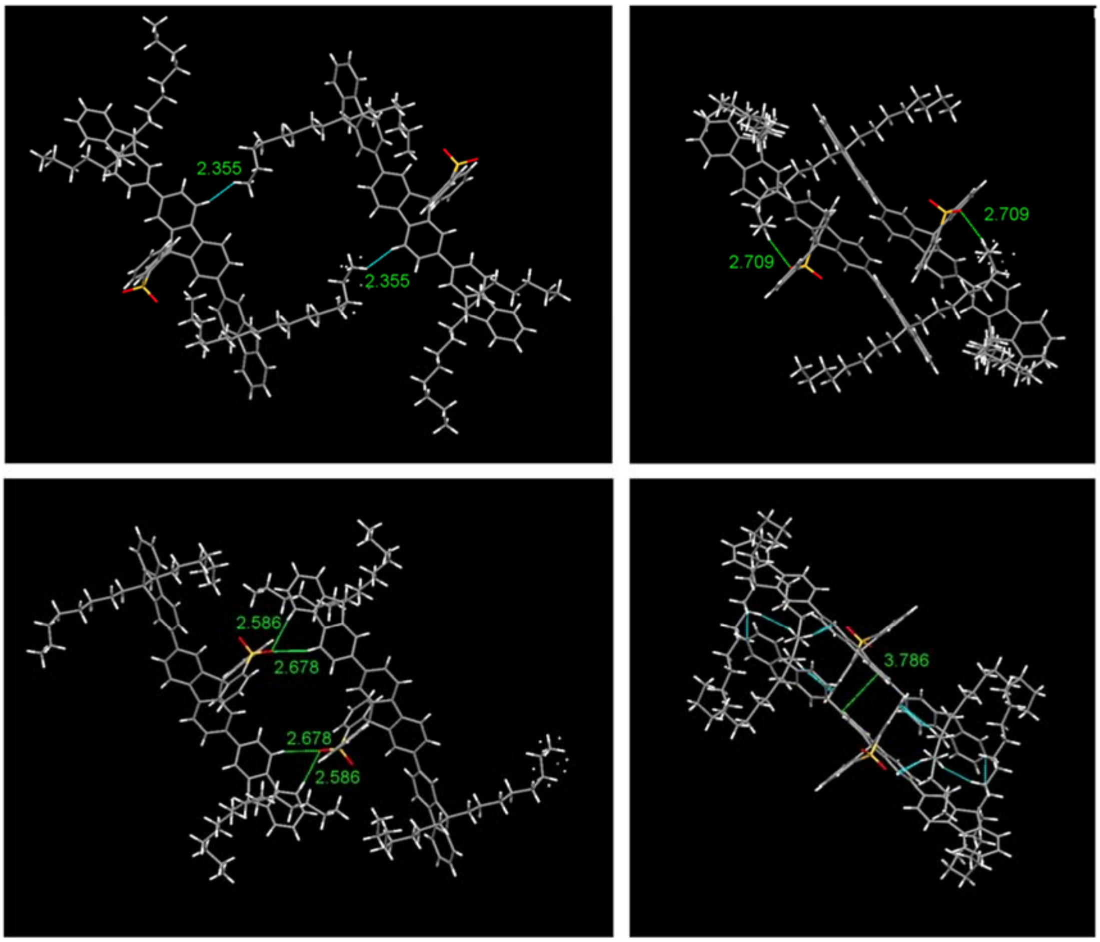

Figure S8. Intermolecular interactions in DOF-SFXSO crystal. Related to Figure 2.

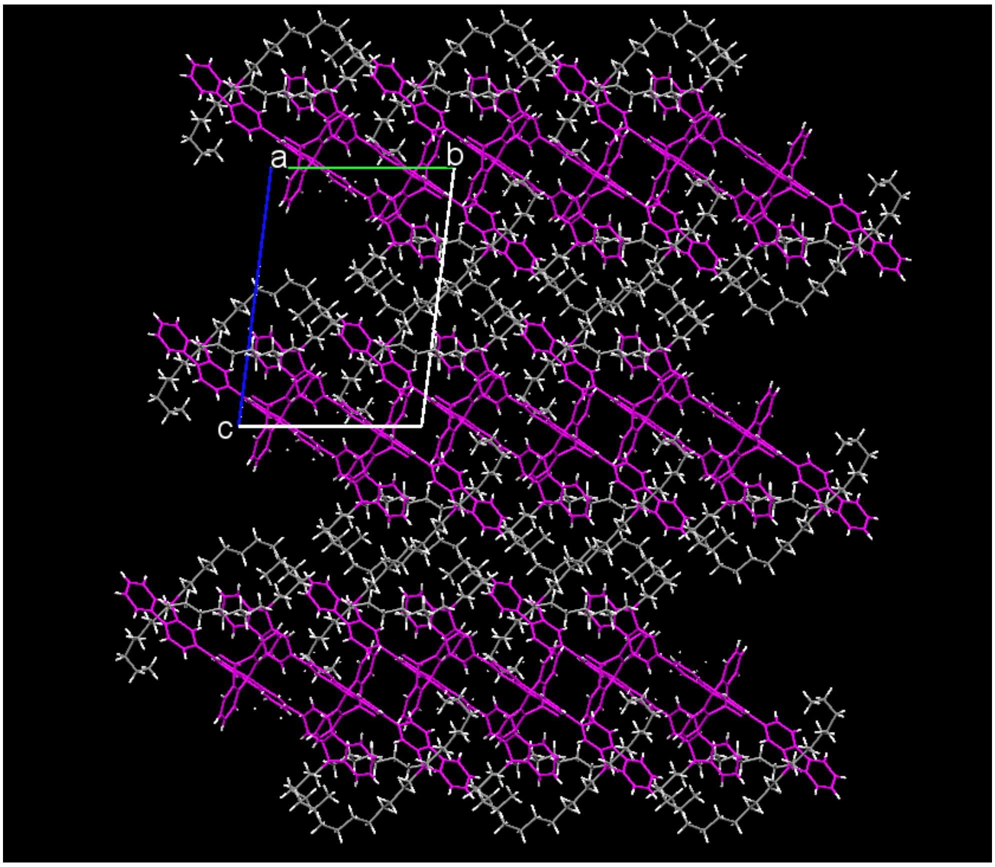

Figure S9. Packing mode of DOF-SFSO crystal view from a-axis. Related to Figure 2.

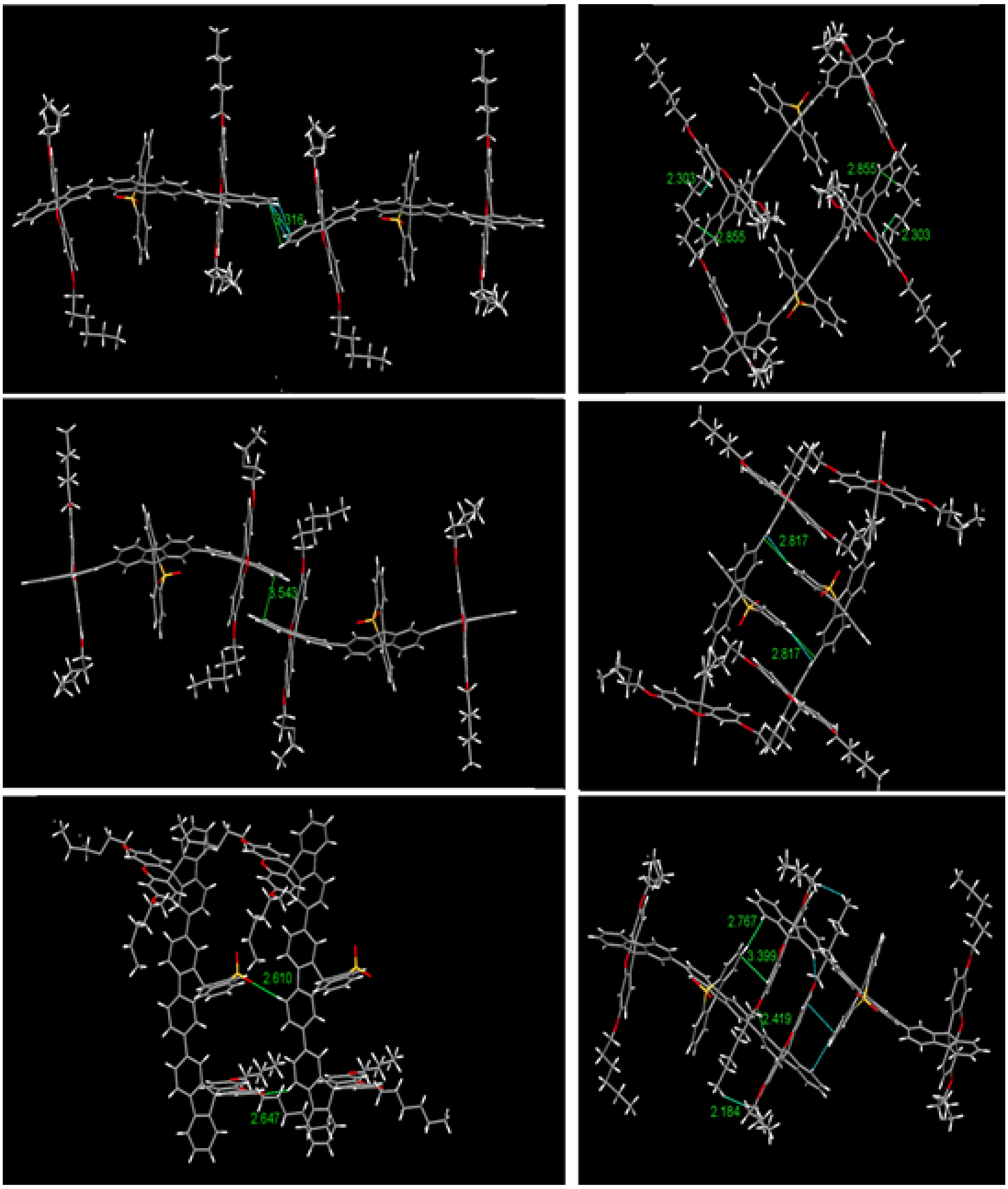

Figure S10. Intermolecular interactions between DHSFX-SFXSO dimers. Related to Figure 2.

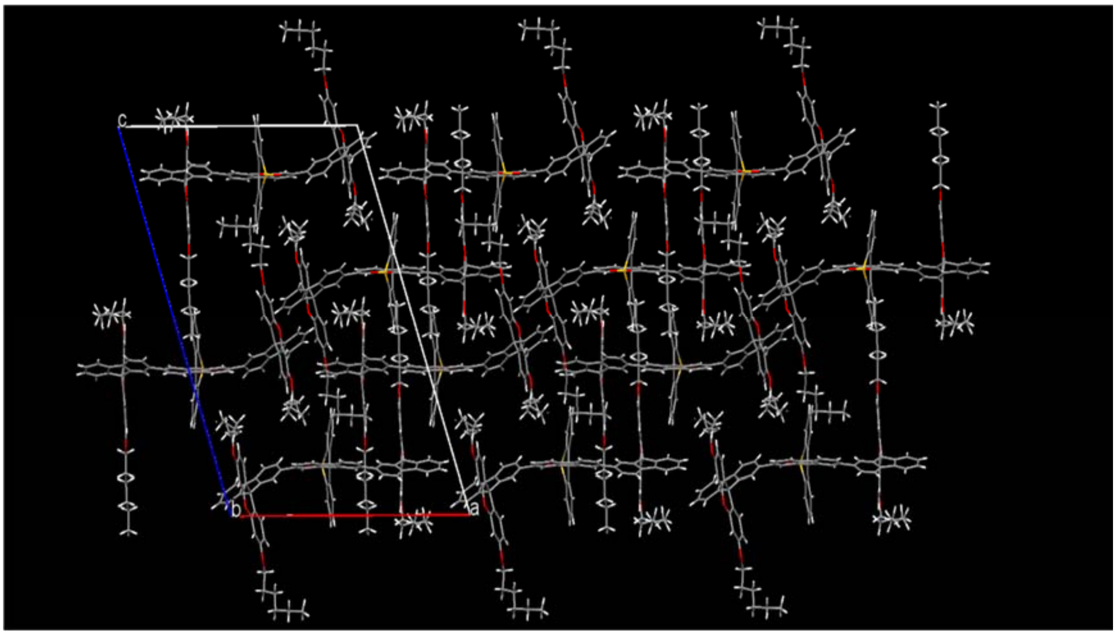

Figure S11. Packing mode of DHSFX-SFSO crystal. Related to Figure 2.

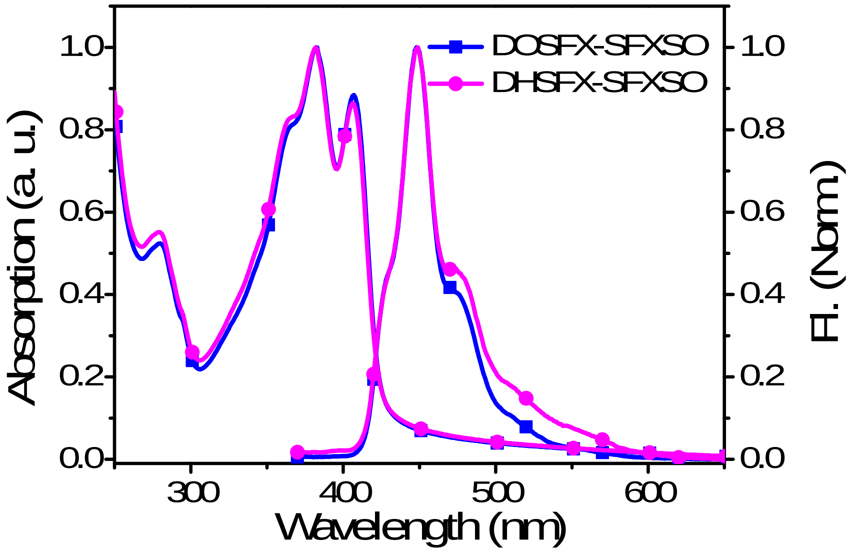

Figure S12. Absorption and PL spectra of DOSFX-SFXSO and DHSFX-SFXSO films after thermal annealing at 180 °C. Related to Figure 1.

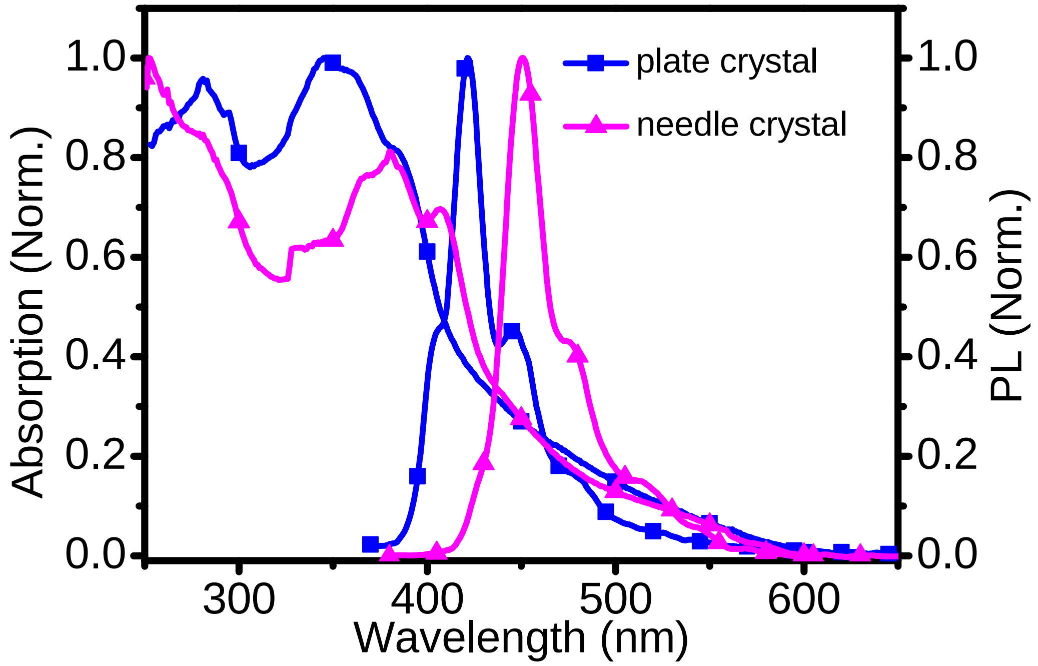

Figure S13. Absorption and PL spectra of two type single crystals of DOSFX-SFXSO. Related to Figure 2.

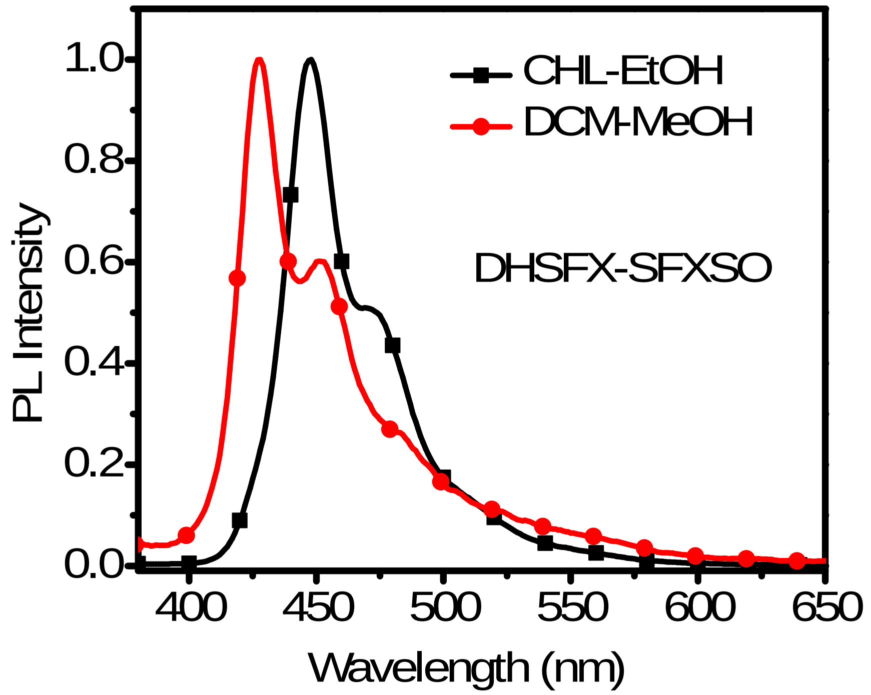

Figure S14. PL spectra of DHSFX-SFXSO microcrystals obtained from chloroform-ethanol and dichloromethane-methanol solutions, respectively. The maximum emission peaks are 427 and 447 nm, respectively. Related to Figure 2.

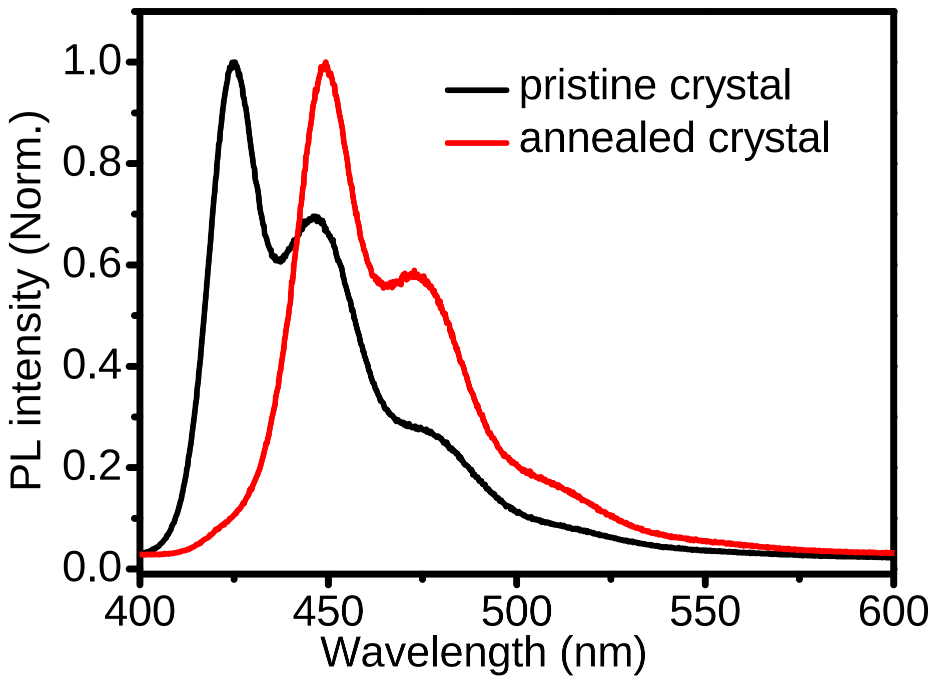

Figure S15. PL spectra of DOSFX-SFXSO pristine flake single crystal and after thermal annealing. Related to Figure 2.

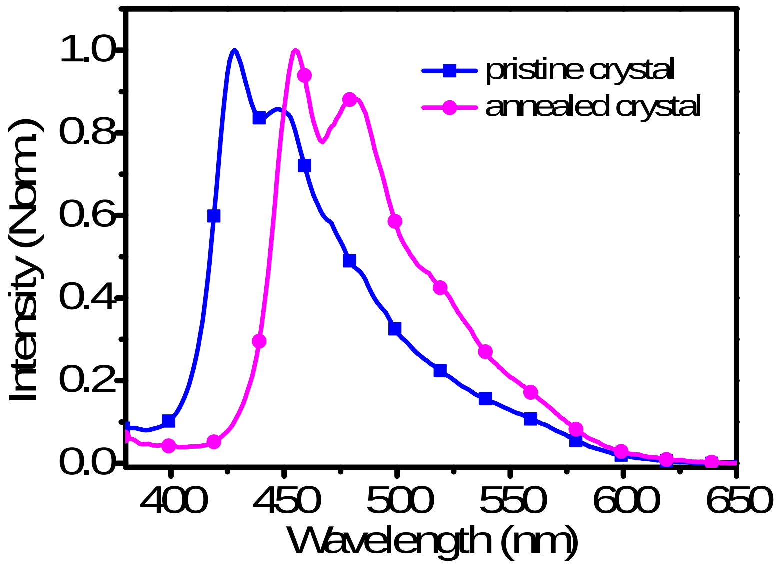

Figure S16. PL spectra of DOSFX-SFXSO pristine flake single crystal and after thermal annealing. Related to Figure 2.

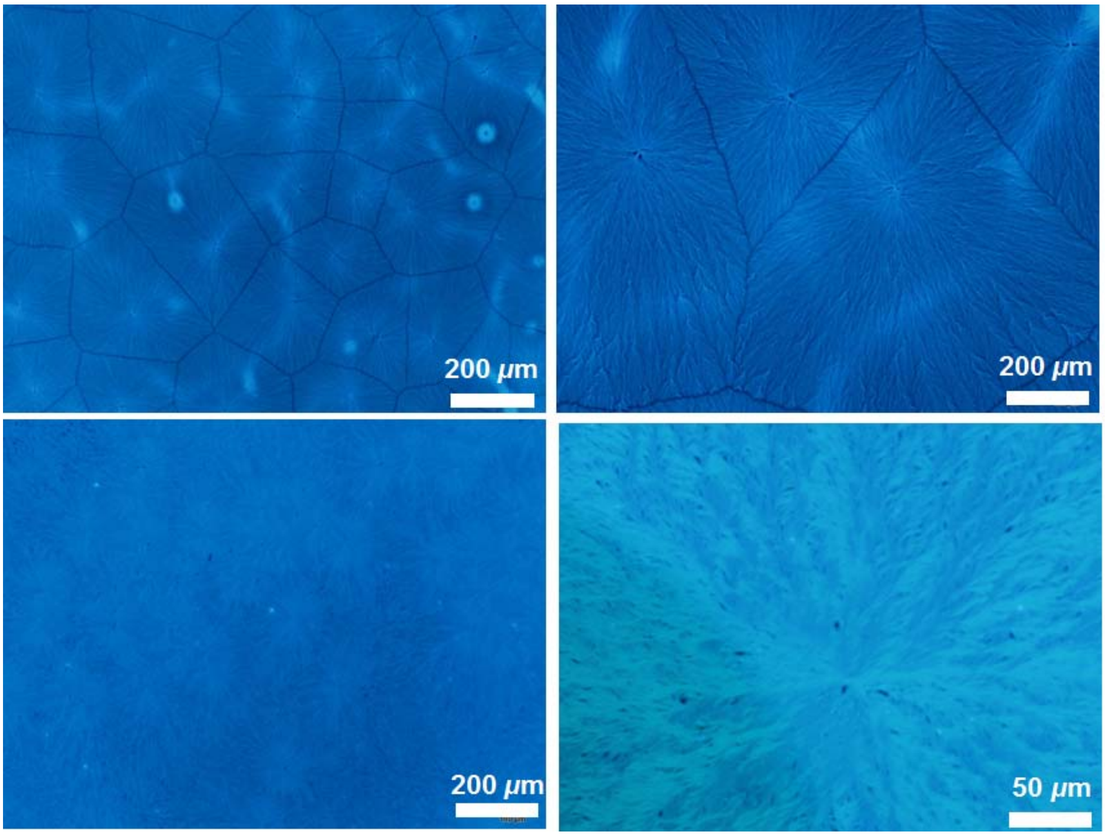

Figure S17. Fluorescence Microscope image of DOSFX-SFXSO spherulites. Related to Figure 2.

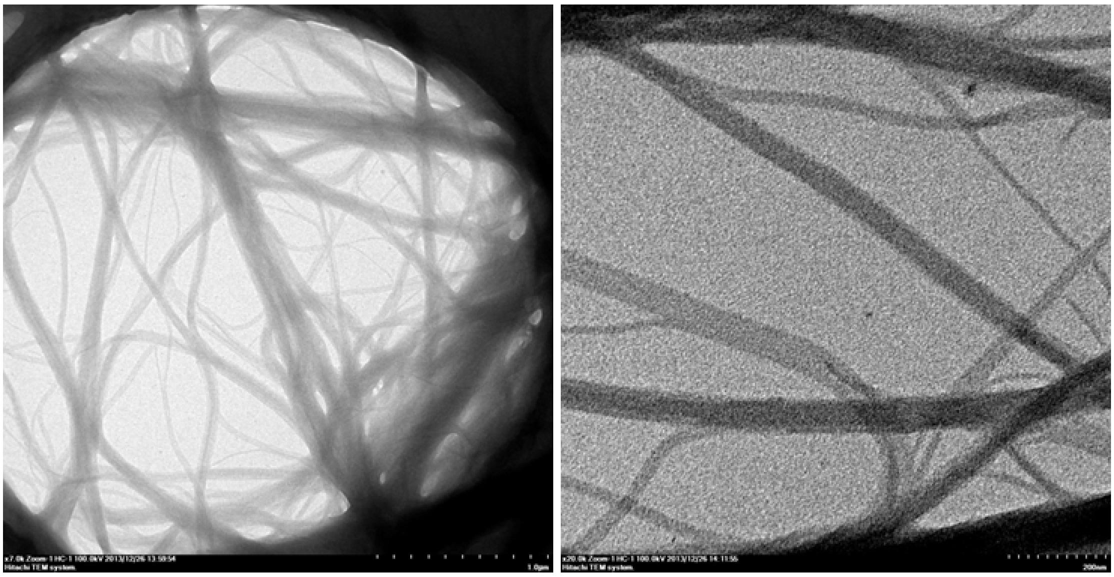

Figure S18. TEM images of DOSFX-SFXSO nanowires. Related to Figure 2.

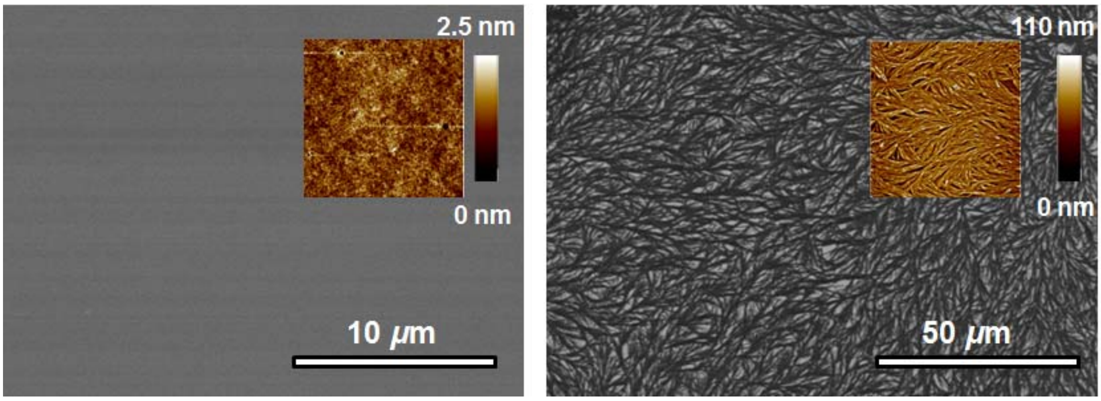

Figure S19. SEM images and its corresponding AFM images (Inset) of DOSFX-SFSO pristine films (Right) and annealed films (Left). Related to Figure 2.

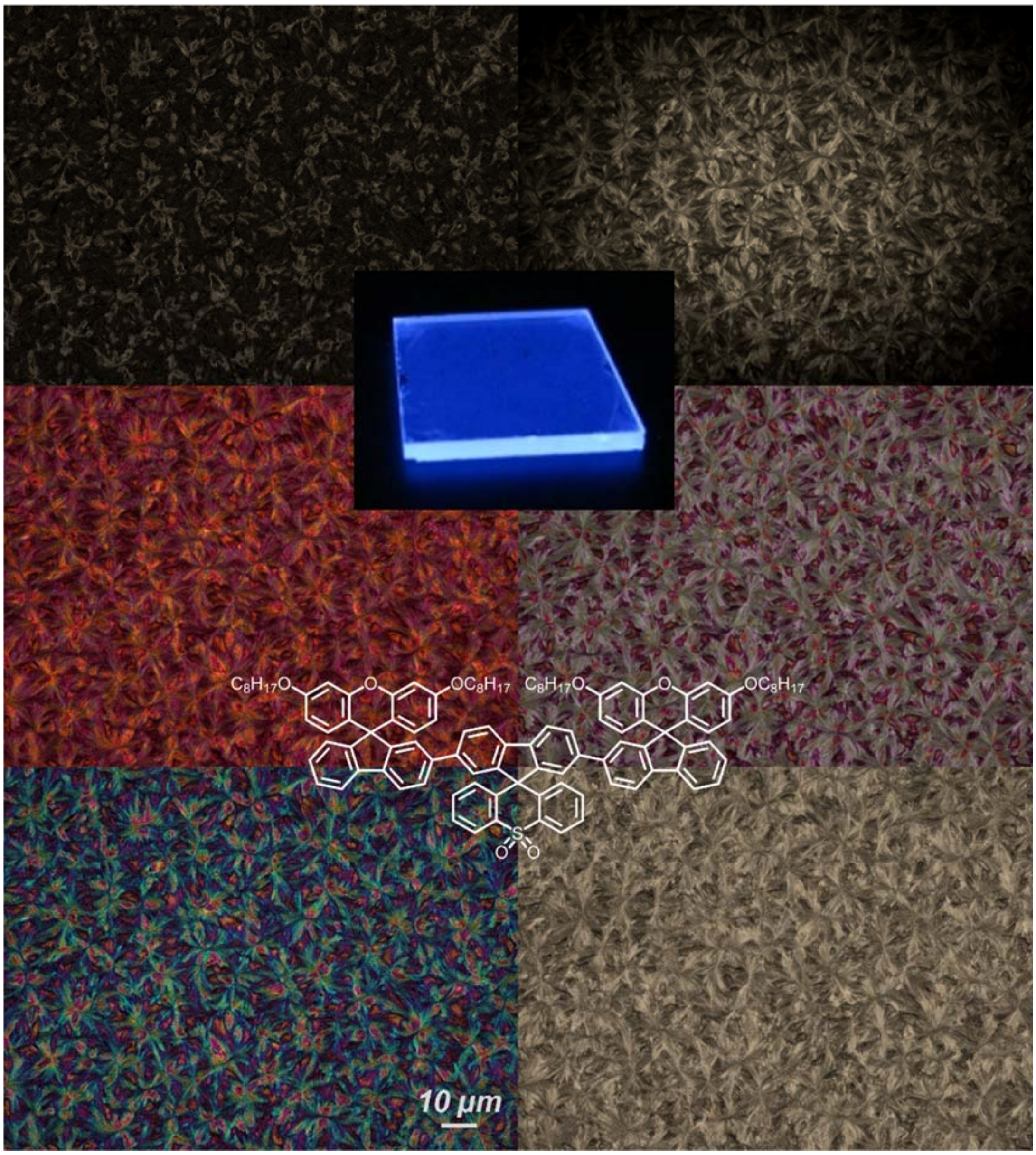

Figure S20. Polarised light images of DOSFX-SFSO annealed films. The same image but with different filters applied, revealing the texture and different regions within the crystal. Related to Figure 2.

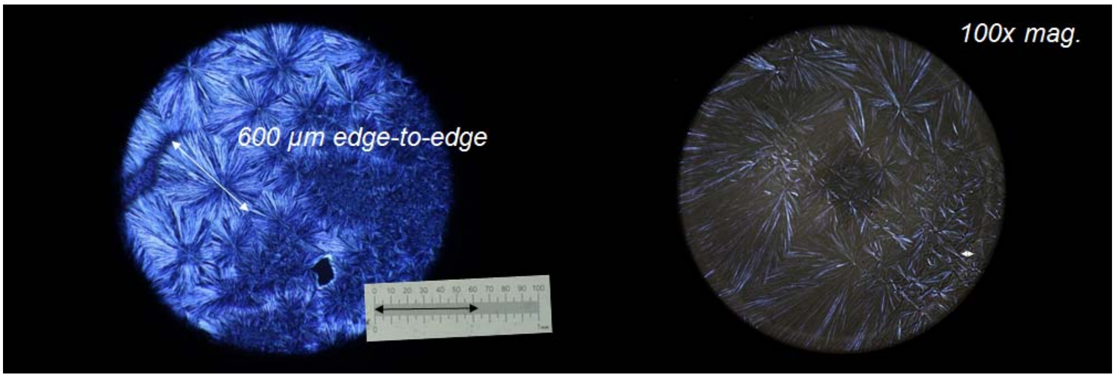

Figure S21. Cross-polarised light microscope image of DOSFX-SFX and DOSFX-SFSO annealed films. Related to Figure 2.

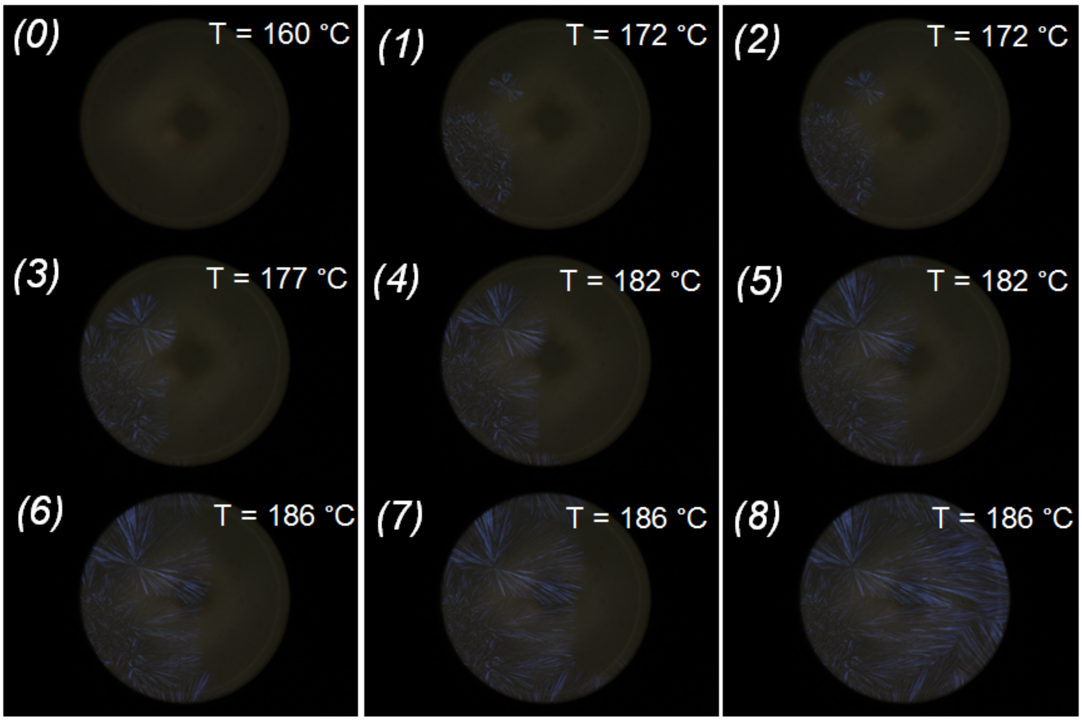

Figure S22. Polarised light microscope image of DOSFX-SFSO annealed films under thermal annealing at various temperatures. Related to Figure 2.

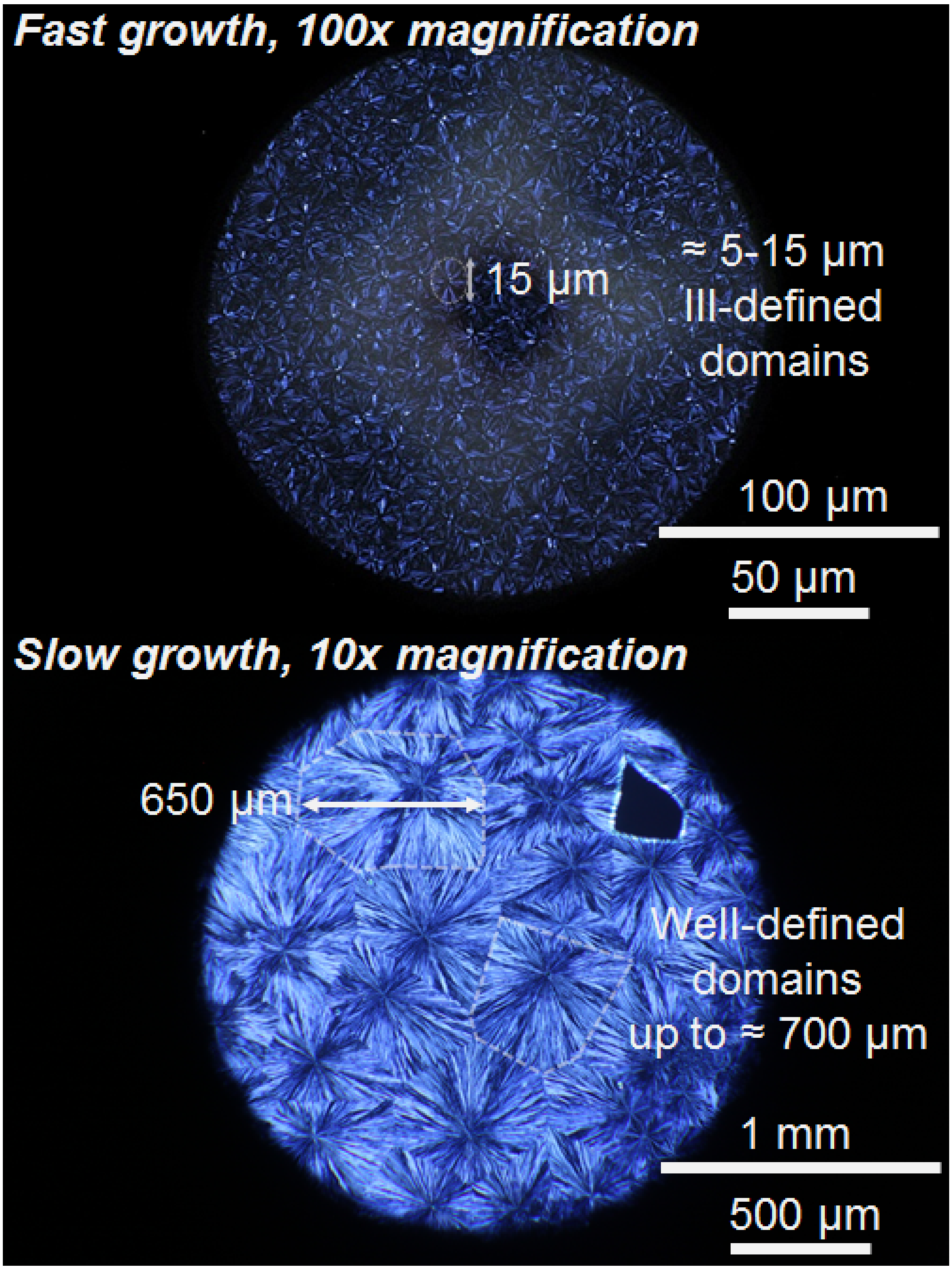

Figure S23. Polarised light microscope image of DOSFX-SFSO annealed films at different crystalline growth ratio. Related to Figure 2.

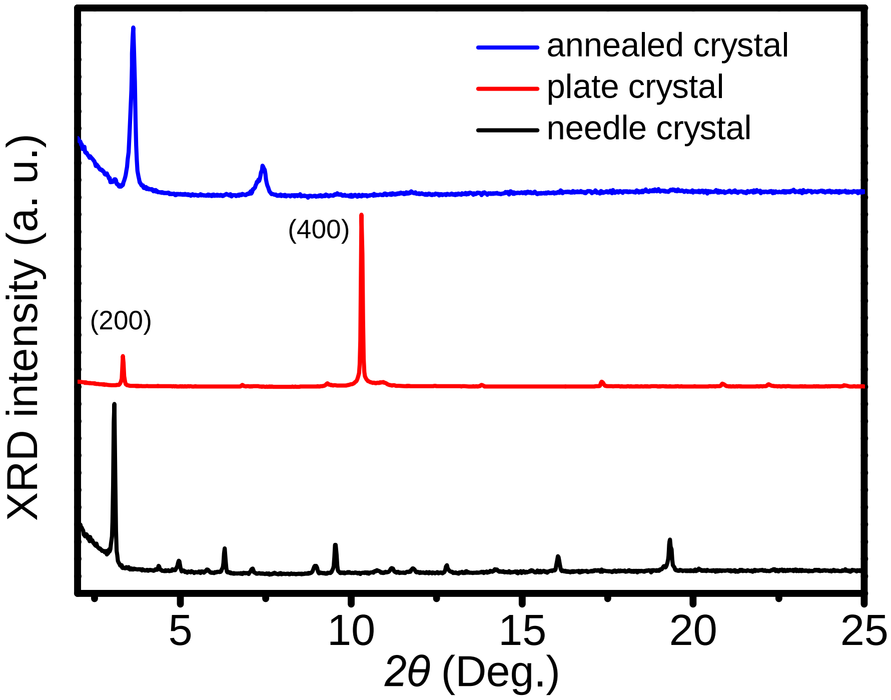

Figure S24. XRD data of DOSFX-SFSO annealed films, plate and needle single crystal. Related to Figure 2.

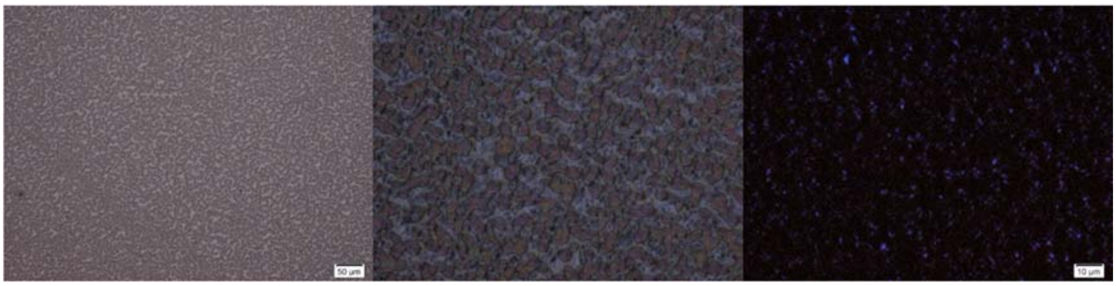

Figure S25. Microscope images of DSFX-OSFX annealed films under non-polarised light, polarized and cross-polarised light. Related to Figure 3.

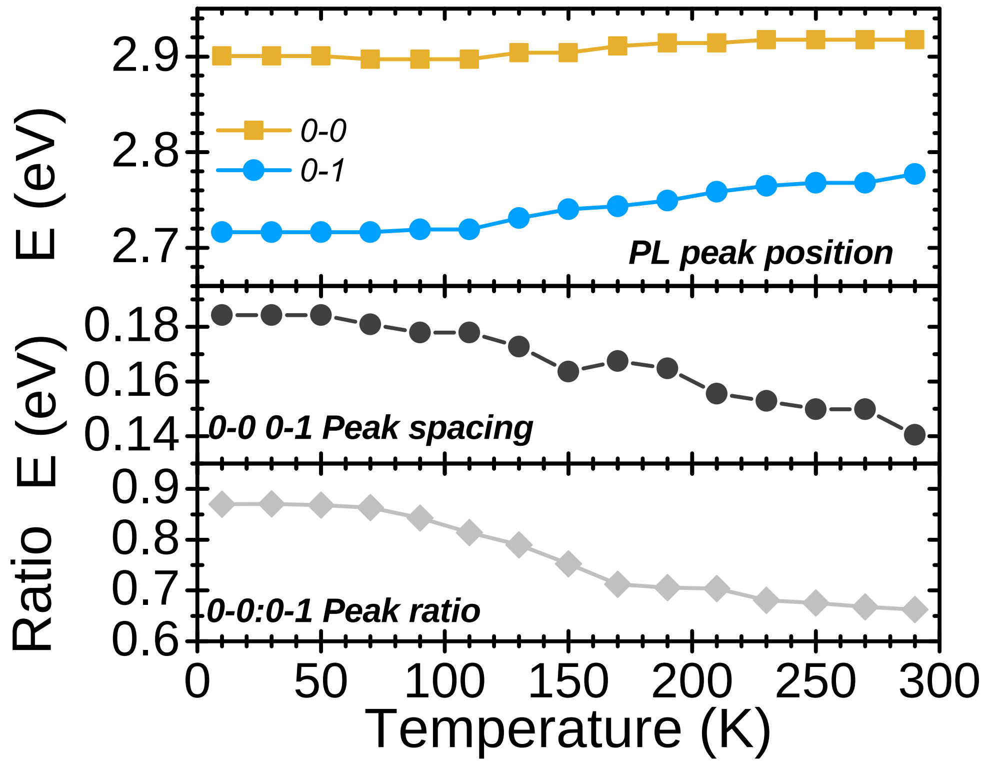

Figure S26. 0-0 - 0-1 peak intensity ratio of DOSFX-SFXSO annealed films upon decreasing temperature from 290 to 10 K. Related to Figure 3.

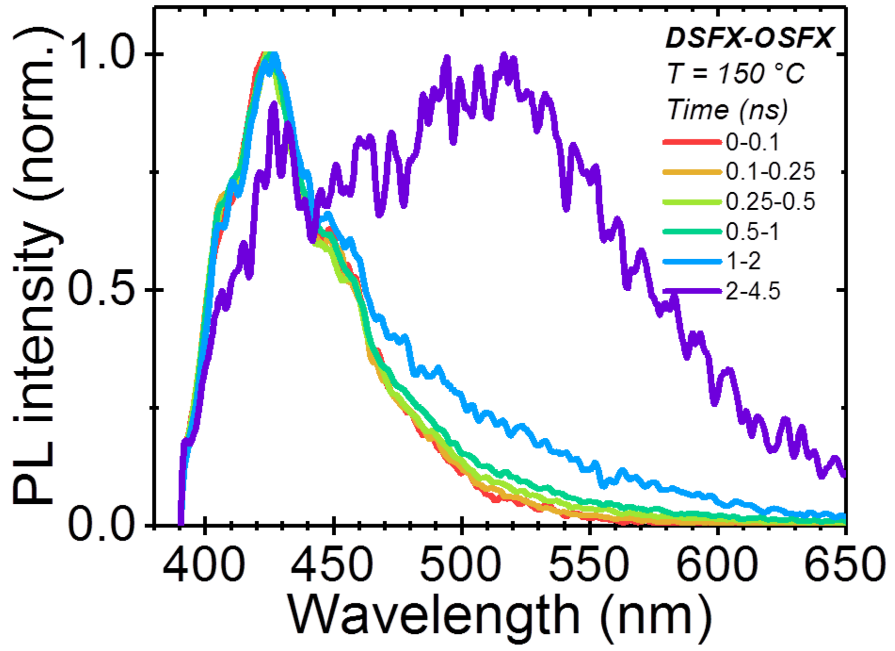

Figure S27. The PL spectra of DSFX-OSFX annealed (crystalline) film excited at 365 nm using a frequency-doubled, mode locked Ti:sapphire laser and measured at 0 ns~4.5 ns following excitation. Related to Figure 3.

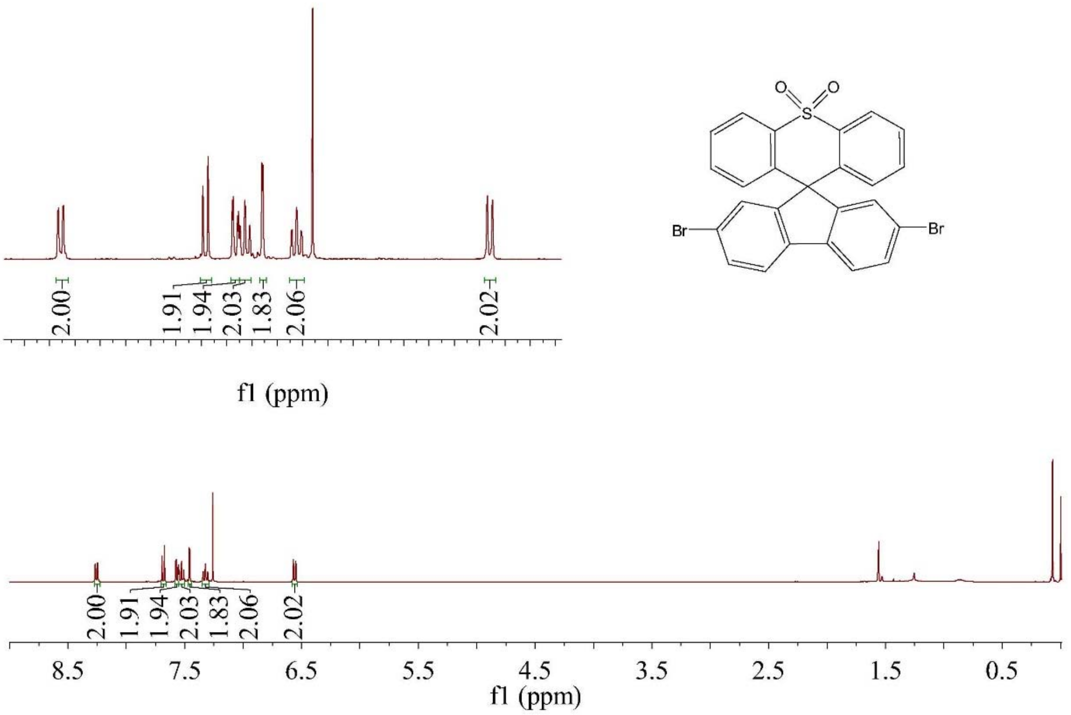

Figure S28. ^{1}H **NMR spectrum of 2,7-dibromospiro[fluorene-9,9'-thioxanthene] 10',10'-dioxide. Related to Figure 1.**

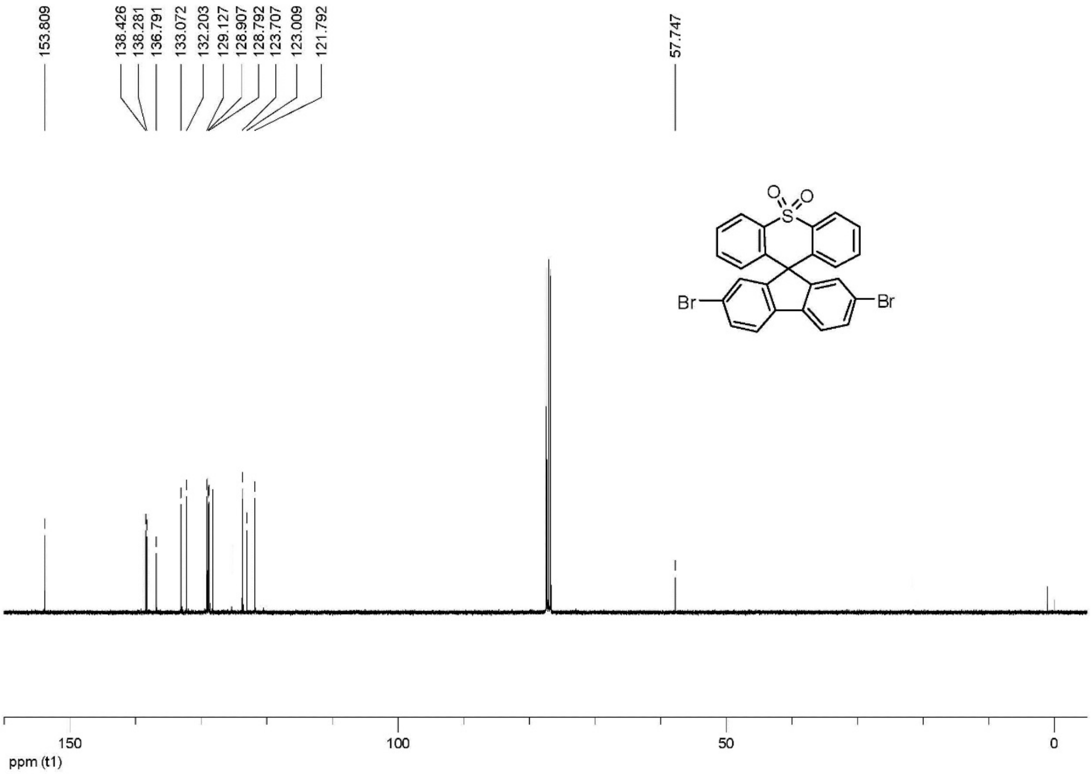

Figure S29. ^{13}C **NMR spectrum of 2,7-dibromospiro[fluorene-9,9'-thioxanthene] 10',10'-dioxide. Related to Figure 1.**

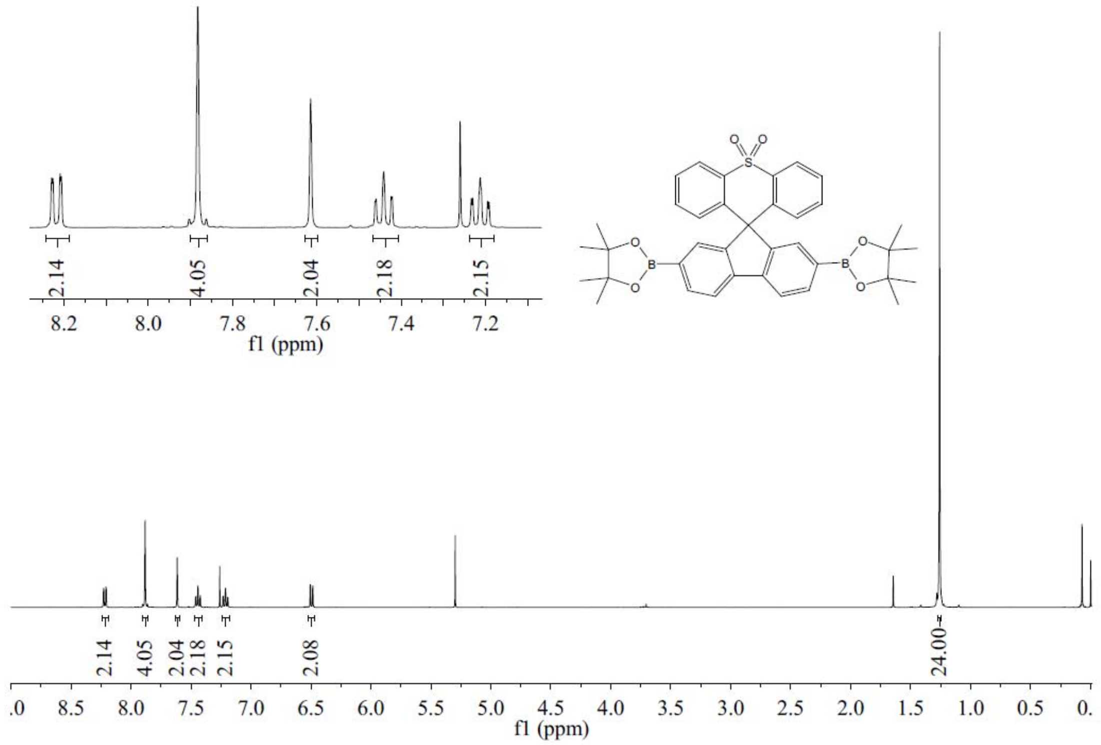

Figure S30. ^{1}H NMR spectrum of 2,7-bis(4,4,5,5-tetramethyl-1,3,2-dioxaborolan-2-yl)spiro[fluorene-9,9'-thioxanthene] 10',10'-dioxide. Related to Figure 1.

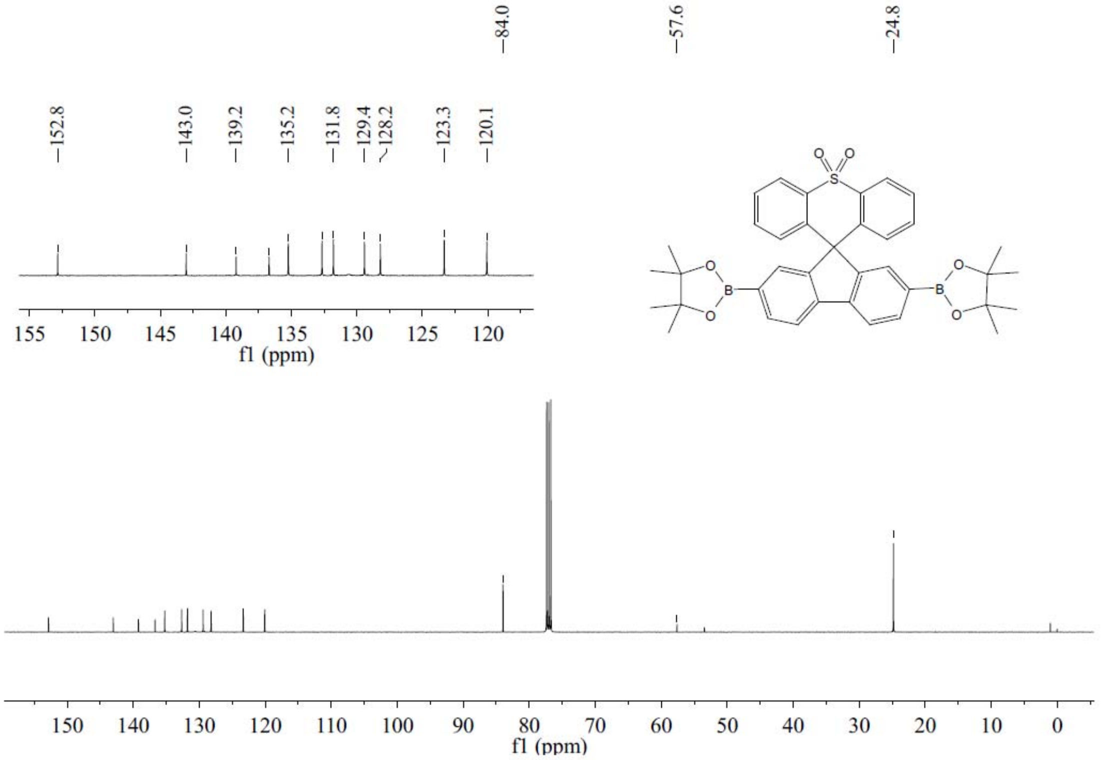

Figure S31. ^{13}C NMR spectrum of 2,7-bis(4,4,5,5-tetramethyl-1,3,2-dioxaborolan

-2-yl)spiro[fluorene-9,9'-thioxanthene] 10',10'-dioxide. Related to Figure 1.

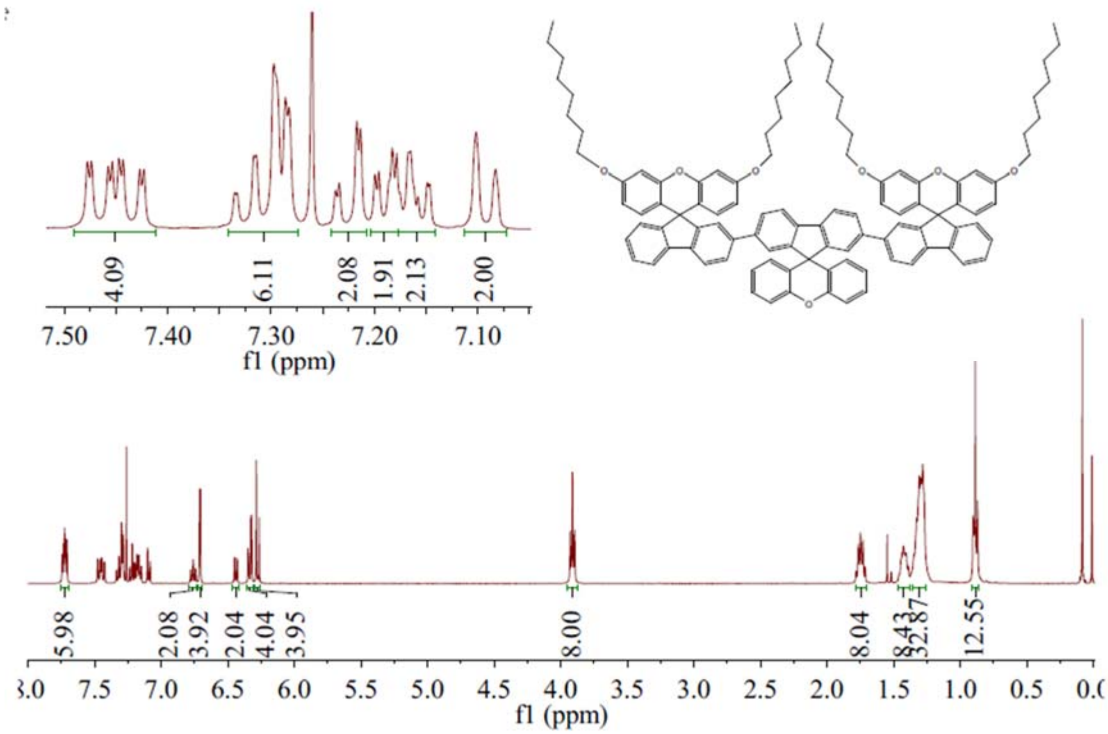

Figure S32. ^{1}H NMR spectrum of DOSFX-SFX. Related to Figure 1.

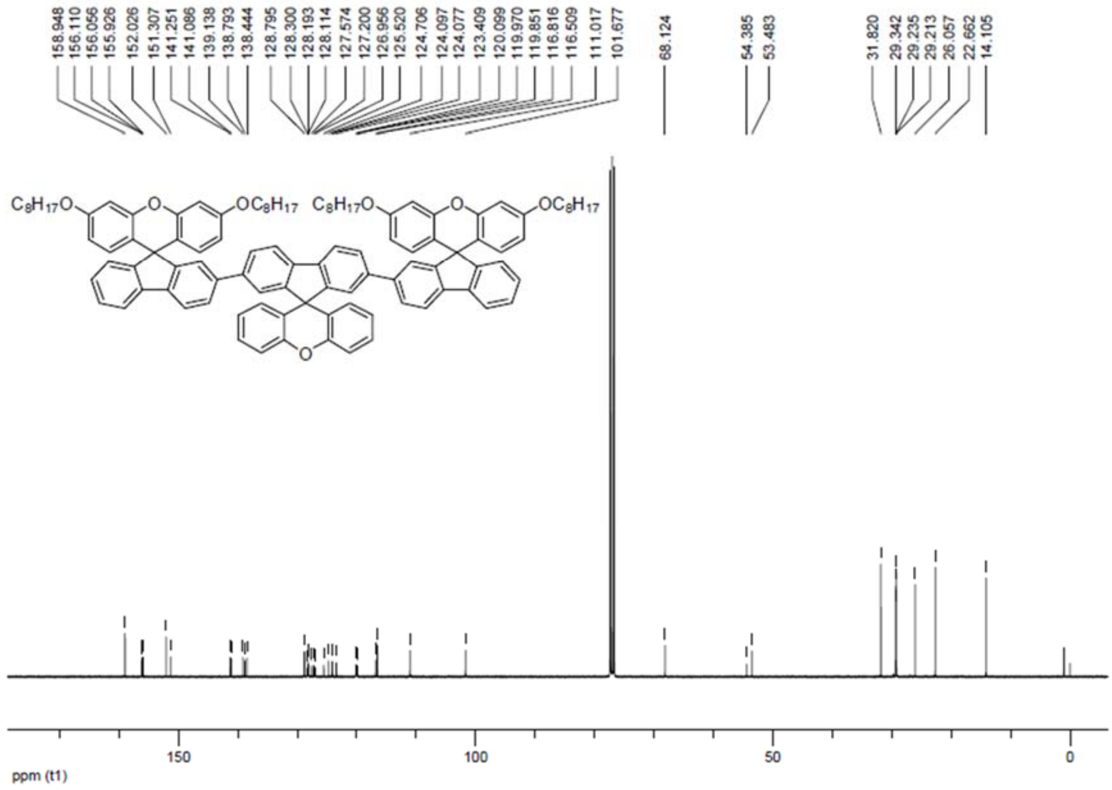

Figure S33. ^{13}C NMR spectrum of DOSFX-SFX. Related to Figure 1.

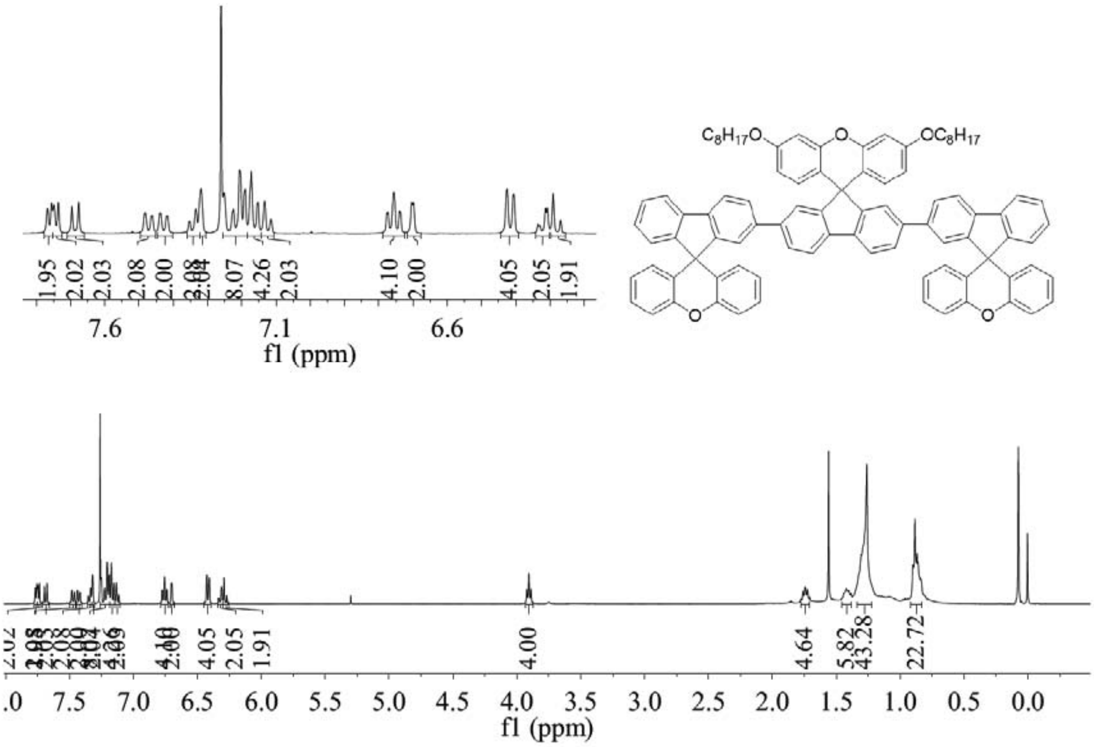

Figure S34. ^{1}H **NMR spectrum of DSFX-OSFX. Related to Figure 1.**

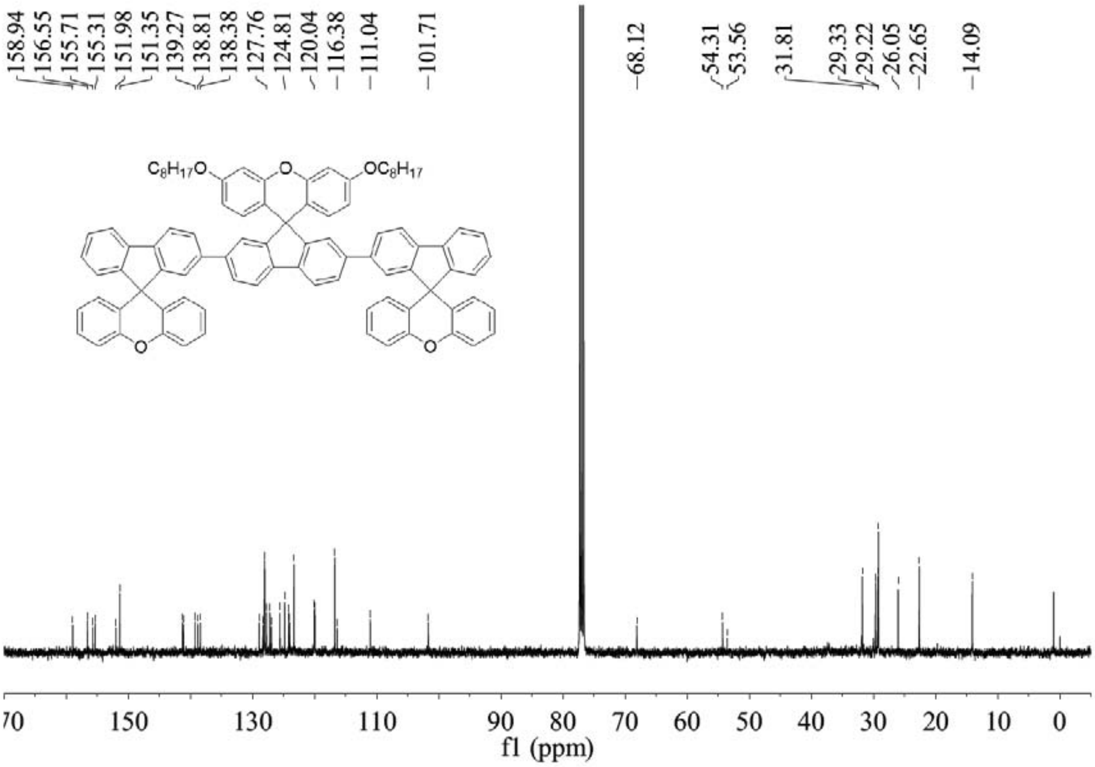

Figure S35. ^{13}C **NMR spectrum of DSFX-OSFX. Related to Figure 1.**

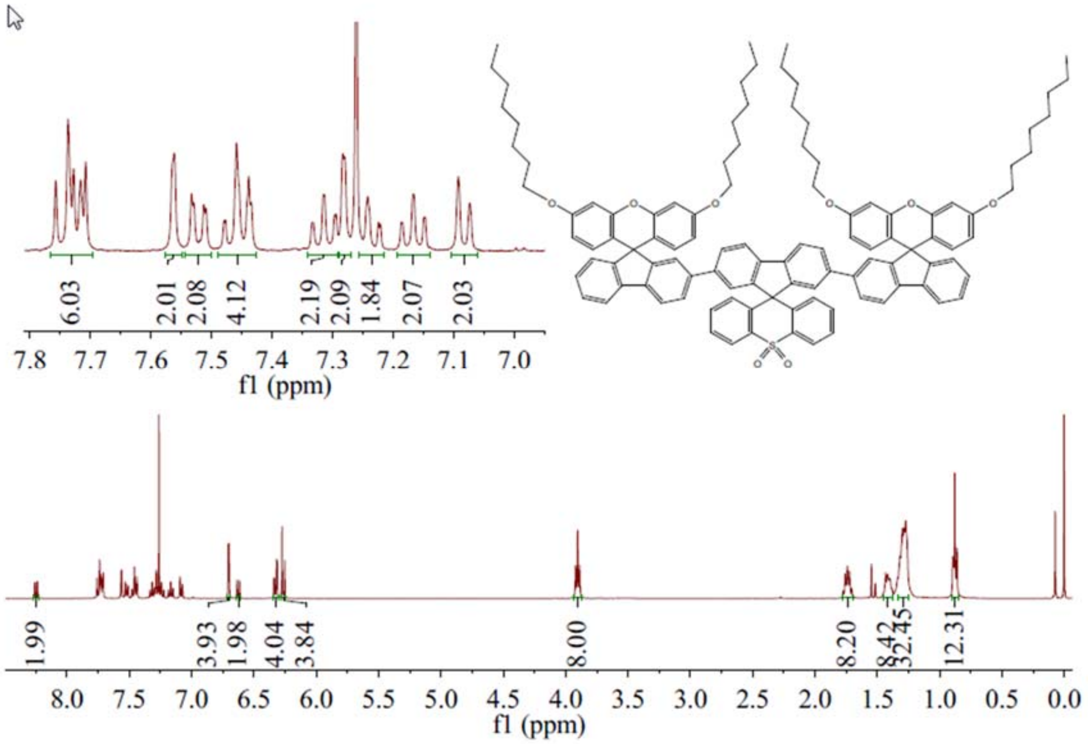

Figure S36. ^{1}H NMR spectrum of DOSFX-SFXSO. Related to Figure 1.

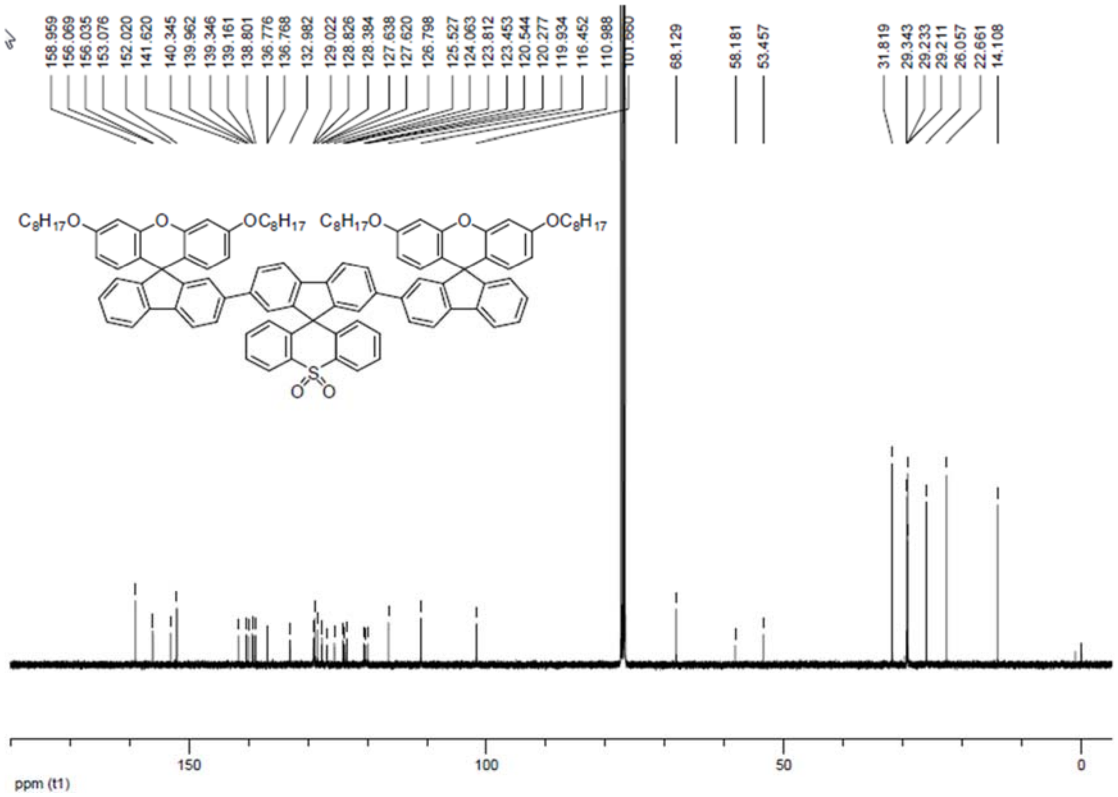

Figure S37. ^{13}C NMR spectrum of DOSFX-SFXSO. Related to Figure 1.

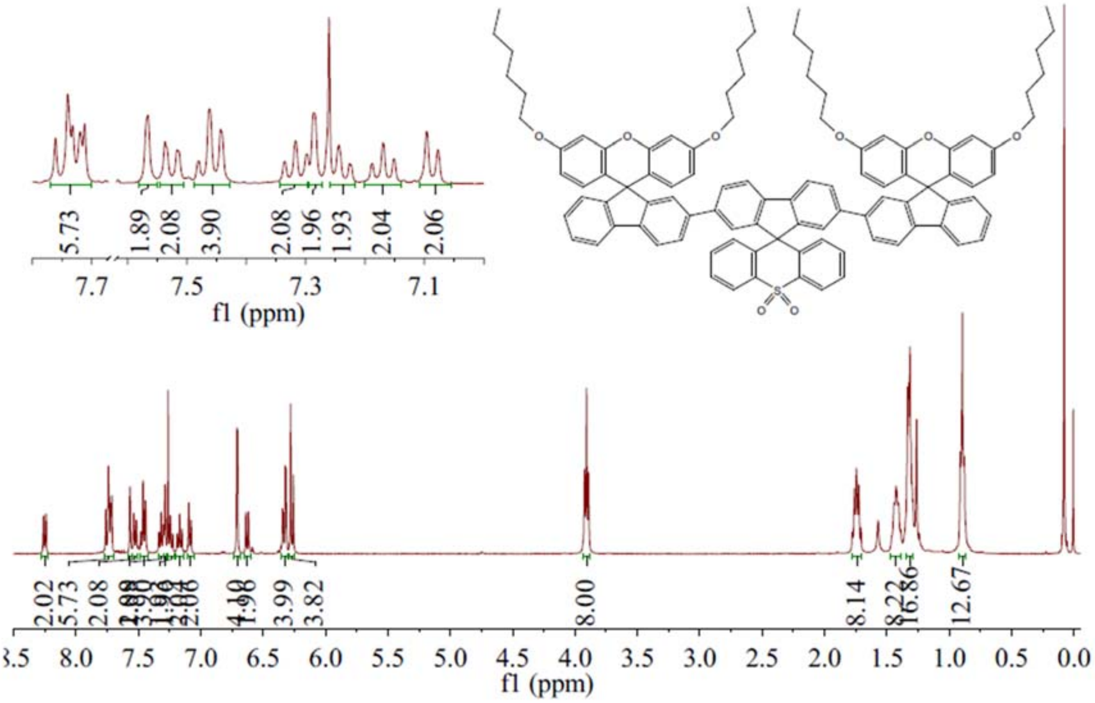

Figure S38. ^{1}H NMR spectrum of DHSFX-SFXSO. Related to Figure 1.

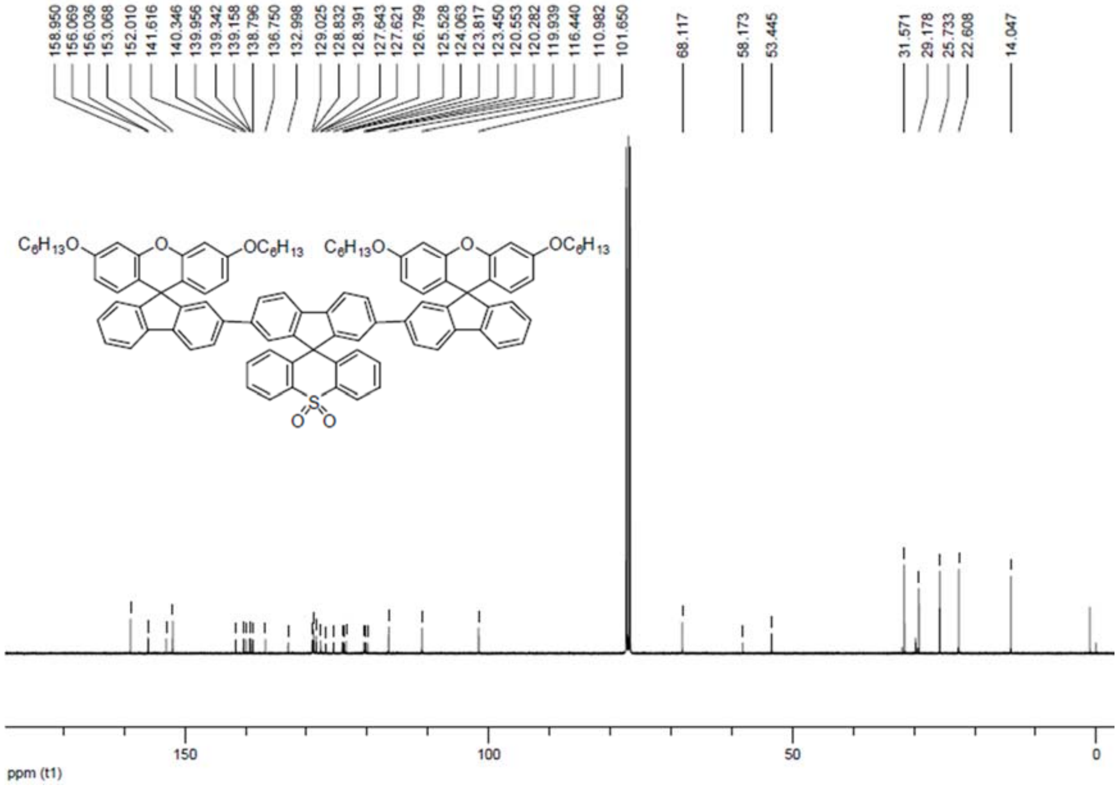

Figure S39. ^{13}C NMR spectrum of DHSFX-SFXSO. Related to Figure 1.

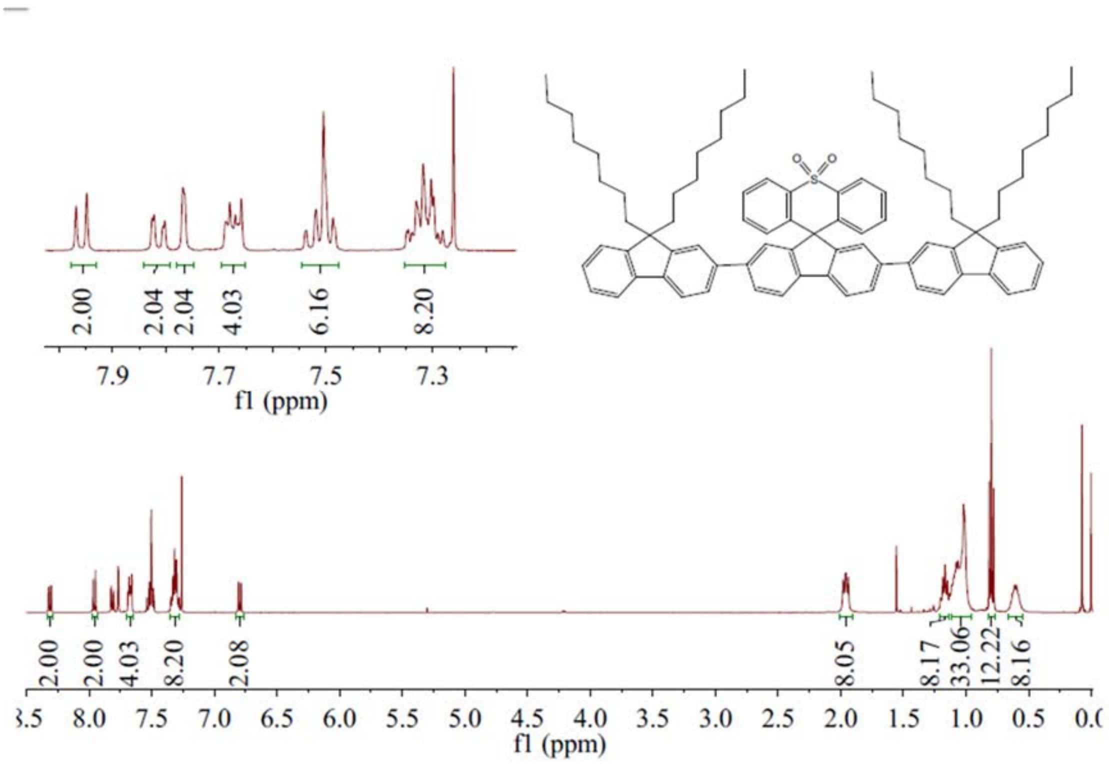

Figure S40. ^{1}**H NMR spectrum of DOF-SFXSO. Related to Figure 1.**

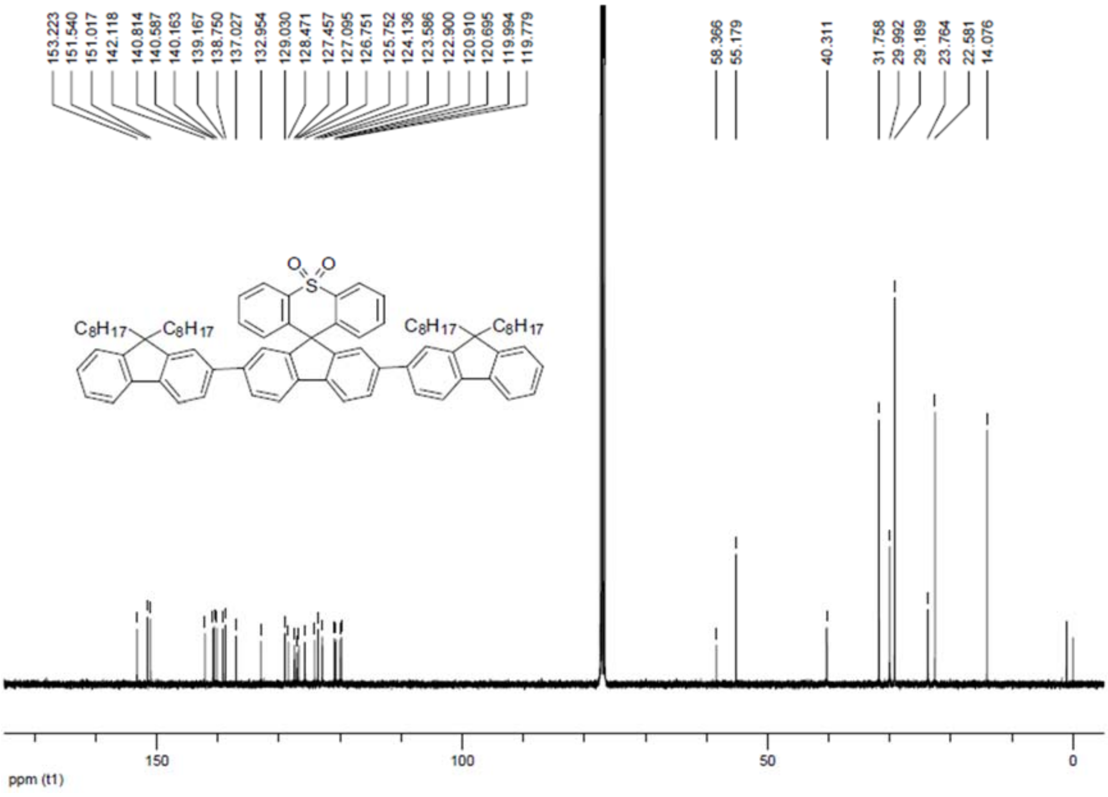

Figure S41. ^{13}**C NMR spectrum of DOF-SFXSO. Related to Figure 1.**

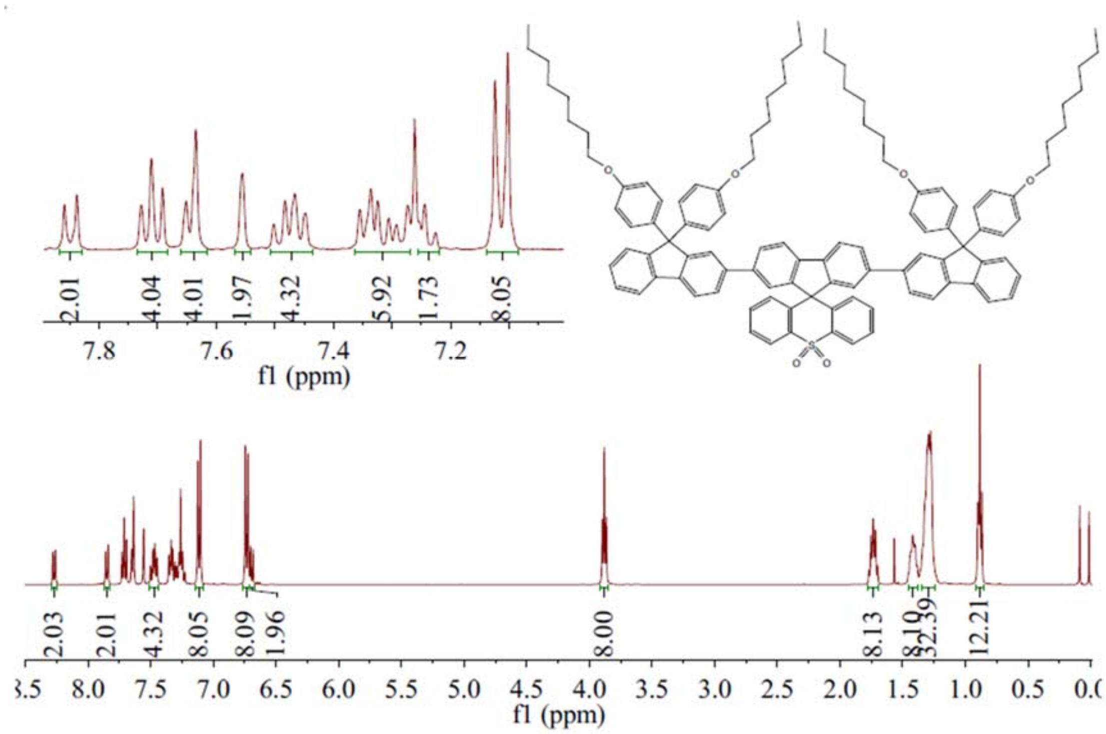

Figure S42. ^{1}H NMR spectrum of DOPhF-SFXSO. Related to Figure 1.

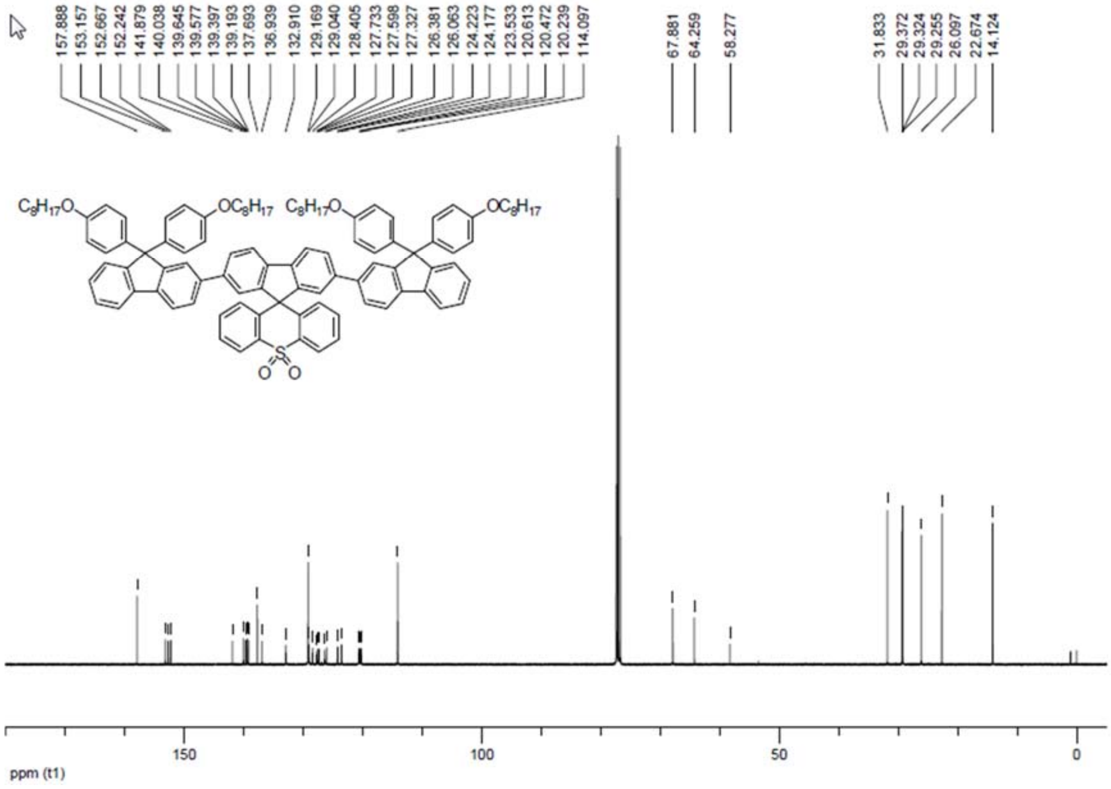

Figure S43. ^{13}C NMR spectrum of DOPhF-SFXSO. Related to Figure 1.

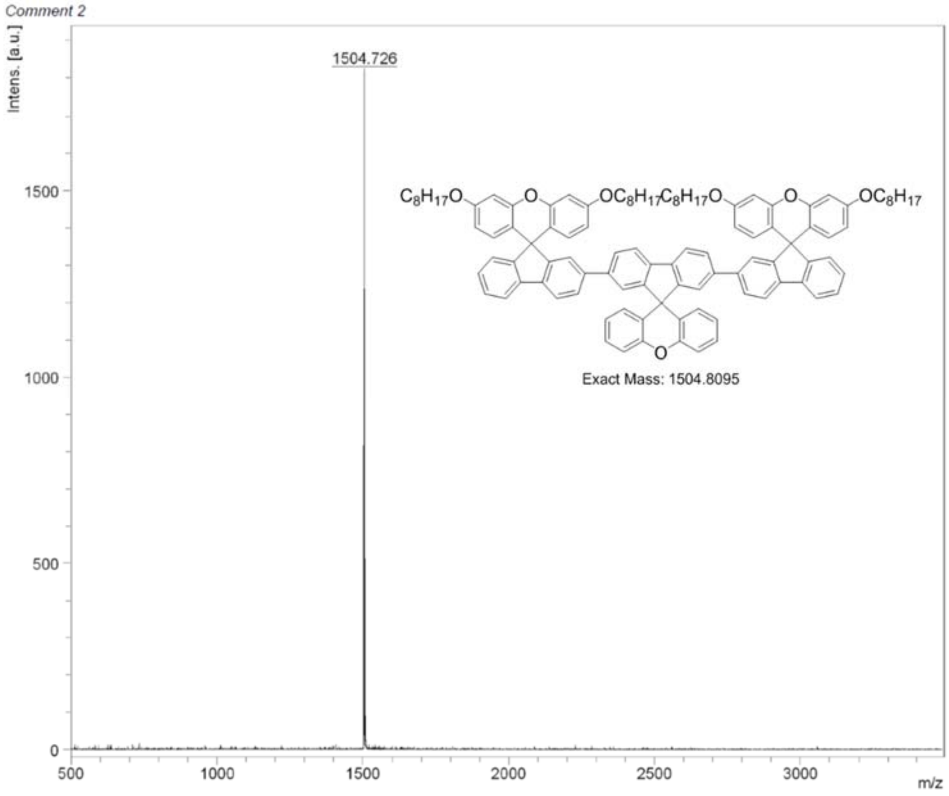

Figure S44. MALDI-TOF mass spectrum of DOSFX-SFX. Related to Figure 1.

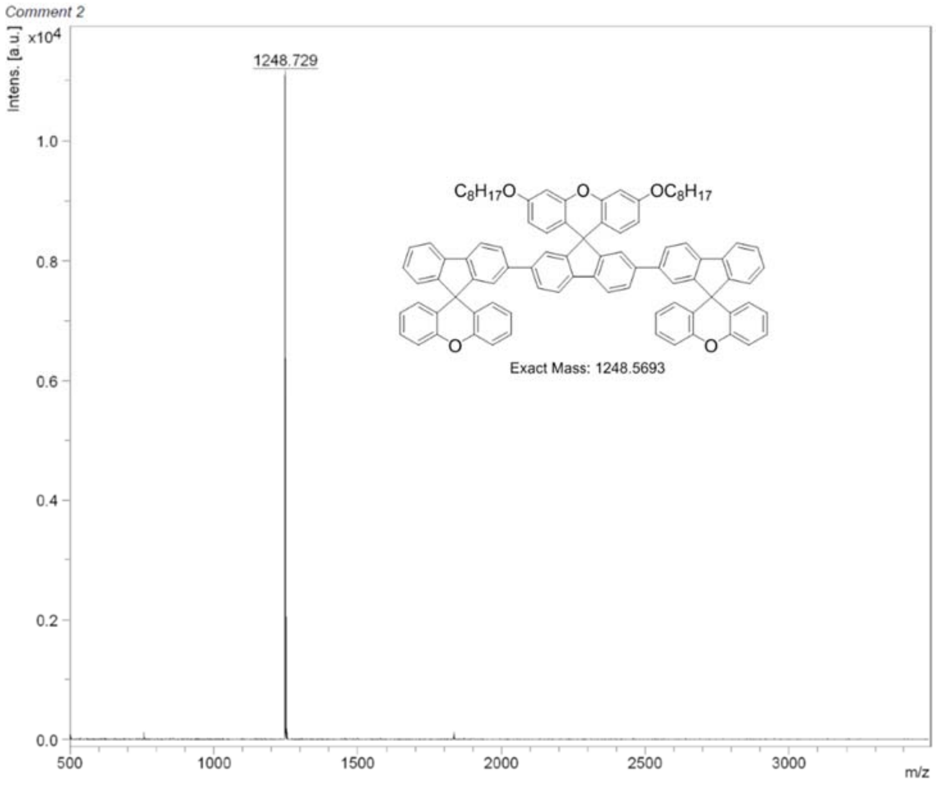

Figure S45. MALDI-TOF mass spectrum of DSFX-OSFX. Related to Figure 1.

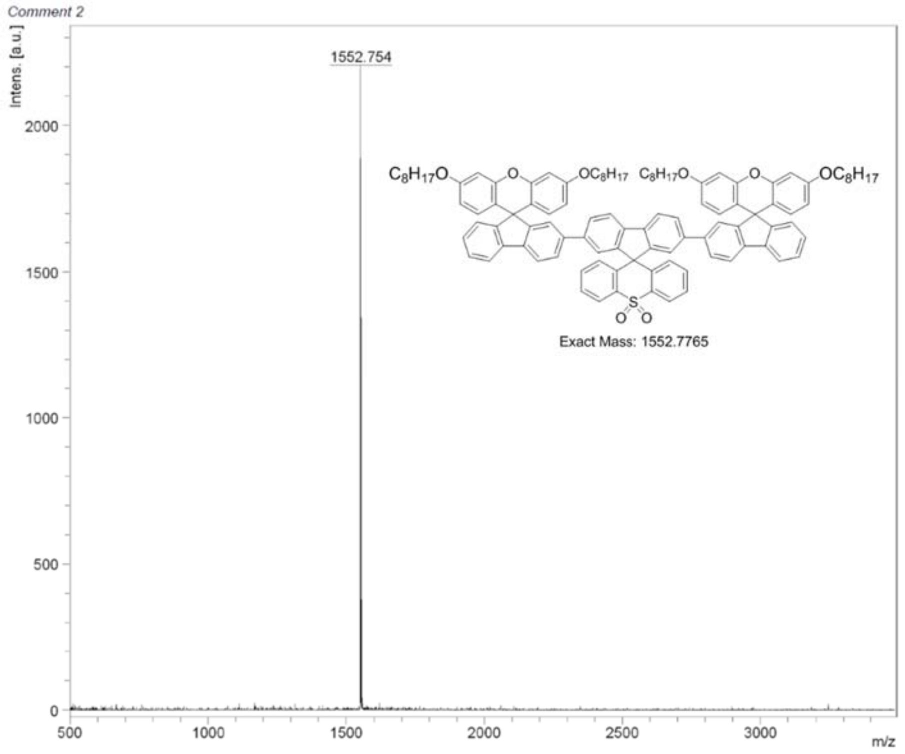

Figure S46. MALDI-TOF mass spectrum of DOSFX-SFXSO. Related to Figure 1.

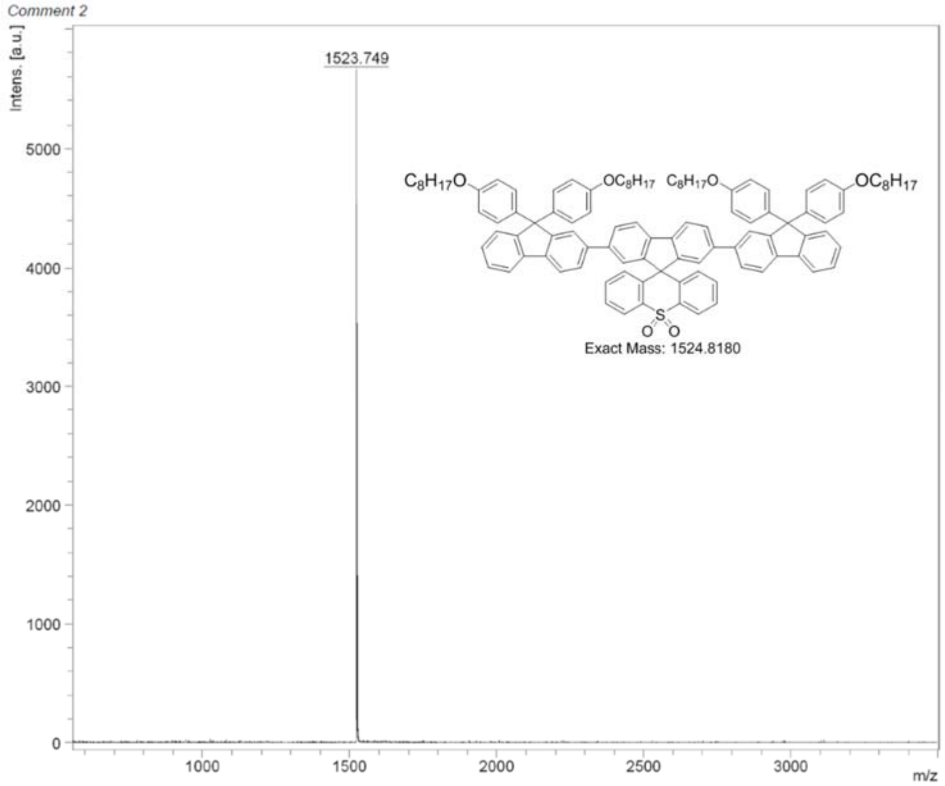

Figure S47. MALDI-TOF mass spectrum of DOPhF-SFXSO. Related to Figure 1.

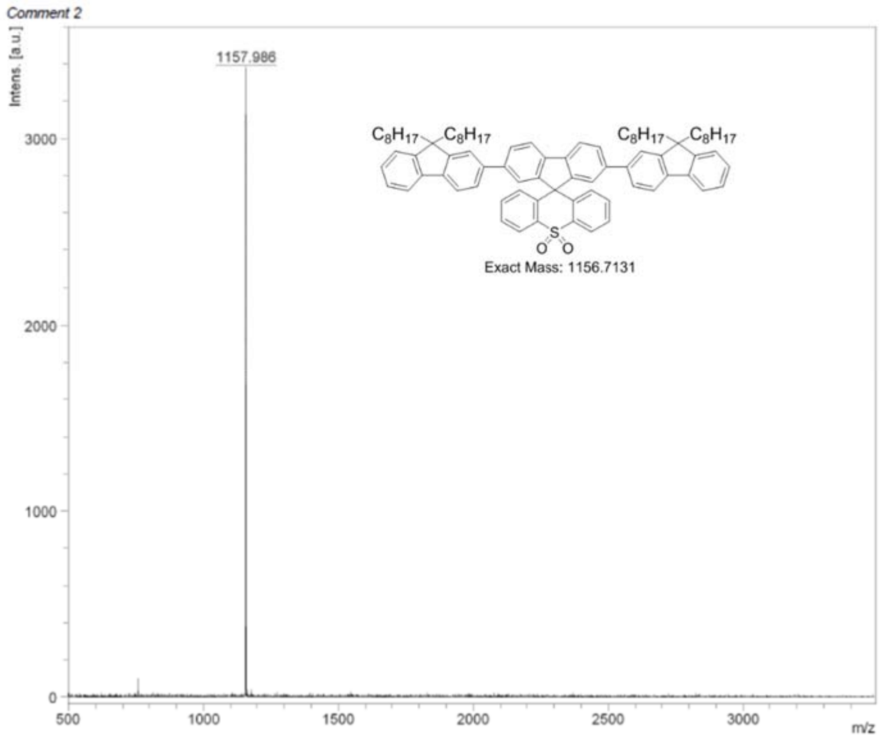

Figure S48. MALDI-TOF mass spectrum of DOF-SFXSO. Related to Figure 1.

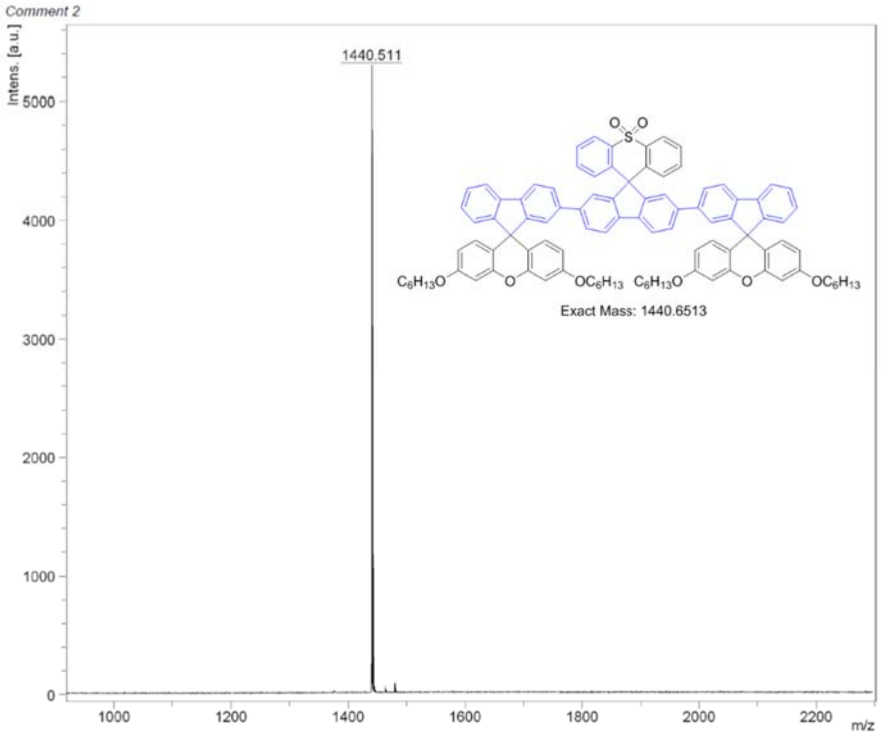

Figure S49. MALDI-TOF mass spectrum of DHSFX-SFXSO. Related to Figure 1.

Table S1. Electrochemical properties of terfluorenes. Related to Figure 1.

Entry	$E_{red,on}$[a] V	$E_{ox,on}$[b] V	LUMO[c] eV	HOMO[d] eV	E_{gap}[e] eV
DOSFX-SFXSO	-2.08	0.93	-2.66	-5.67	3.01
DOSFX-SFX	-2.20	0.89	-2.54	-5.63	3.09
DOF-SFXSO	-2.10	0.86	-2.64	-5.60	2.96
DOPhF-SFXSO	-2.13	0.88	-2.61	-5.62	3.01
DSFX-OSFX	-2.16	0.90	-2.58	-5.64	3.06
DHSFX-SFXSO	-2.05	0.98	-2.69	-5.72	3.03

[a]On-set reduction potential. [b]On-set oxidation potential. [c]LUMO = $-(E_{red,on}-E_{\varphi})-4.80$ eV, where E_{φ} is potential of ferrocene. [d]HOMO = $-(E_{ox,on}-E_{\varphi})-4.80$ eV. [e]E_{gap} = LUMO – HOMO.

Table S2. Crystal data of bulk terfluorenes. Related to Figure ~~1~~2.

name	DOSFX-SFX	DOF-SFXSO	DOSFX-SFXSO	DHSFX-SFXSO
CCDC No.	1448974	1860090	1887107	1887099
formula	$C_{107}H_{108}O_7$	$C_{83}H_{96}O_2S$	$C_{111}H_{112}Cl_2O_8S$	$C_{99}H_{92}O_8S$
fw[g/mol]	1505.93	1157.65	2031.46	1441.78
crystal color	colorless	colorless	colorless	colorless
size [mm]	0.23*0.20*0.15	0.31*0.12*0.07	0.30*0.15*0.05	0.14*0.11*0.06
T [K]	100	173	173	173
lattice type	monoclinic	triclinic	monoclinic	monoclinic
space group	$P2_1/c$	*P*-1	*C*2/c	*P*2/n
a [Å]	18.8804(15)	12.5322 (9)	55.433 (3)	24.7839 (15)
b [Å]	10.6661(9)	13.8332 (11)	10.9061 (5)	10.9378 (6)
c [Å]	42.297(3)	20.1569 (16)	16.7183 (8)	32.4523 (18)
α [°]	90	96.504 (5)	90	90
β [°]	105.485(3)	102.145 (4)	94.057 (4)	110.920 (3)
γ [°]	90	92.417 (4)	90	90
V [Å^3]	8208.59 (11)	3386.4 (5)	10081.8 (38)	8217.3 (8)
Z	4	2	4	4
F(000)	3224	1252	4248	3064
absorption coefficient [mm^{-1}]	0.074	0.774	3.663	0.755
measured	14456	9032	7083	9919
observed	11340	11545	8289	14355
θ range [°]	1.0-25.0	3.22-65.17	3.20-63.87	2.92-66.38
R1	0.0588	0.1033	0.1689	0.0868
ωR2	0.1364	0.2851	0.4260	0.2502
completeness	0.999	0.987	0.991	0.986
S	1.049	1.039	1.049	1.024

Supplemental References

C. J. Ou, C. Zhu, X. H. Ding, L. Yang, J. Y. Lin, L. Xie, Y. Qian, C. Xu, J. F. Zhao and W. Huang, (2017). Dimerization effect of fluorene-based semiconductors on conformational planarization for microcrystal lasing. J. Mater. Chem. C, 5, 5345-5355.

C. J. Ou, X. H. Ding, Y. X. Li, C. Zhu, M. N. Yu, L. Xie, J. Y. Lin, C. Xu and W. Huang, (2017). Conformational effect of polymorphic terfluorene on photophysics, crystal morphologies, and lasing behaviors. J. Phys. Chem. C, 121, 14803-14810.

Z. Zuo, C. Ou, Y. Ding, H. Zhang, S. Sun, L. Xie, R. Xia and W. Huang, (2018). Spiro-substitution effect of terfluorenes on amplified spontaneous emission and lasing behaviors. J. Mater. Chem. C, 2018, 6, 4501-4507.
